# Association Between Smoking and Molecular Subtypes of Colorectal Cancer

**DOI:** 10.1093/jncics/pkab056

**Published:** 2021-06-14

**Authors:** Xiaoliang Wang, Efrat Amitay, Tabitha A Harrison, Barbara L Banbury, Sonja I Berndt, Hermann Brenner, Daniel D Buchanan, Peter T Campbell, Yin Cao, Andrew T Chan, Jenny Chang-Claude, Steven J Gallinger, Marios Giannakis, Graham G Giles, Marc J Gunter, John L Hopper, Mark A Jenkins, Yi Lin, Victor Moreno, Reiko Nishihara, Polly A Newcomb, Shuji Ogino, Amanda I Phipps, Lori C Sakoda, Robert E Schoen, Martha L Slattery, Mingyang Song, Wei Sun, Steven N Thibodeau, Amanda E Toland, Bethany Van Guelpen, Michael O Woods, Li Hsu, Michael Hoffmeister, Ulrike Peters

**Affiliations:** Public Health Sciences Division, Fred Hutchinson Cancer Research Center, Seattle, WA, USA; Department of Epidemiology, University of Washington, Seattle, WA, USA; Division of Clinical Epidemiology and Aging Research, German Cancer Research Center (DKFZ), Heidelberg, Germany; Public Health Sciences Division, Fred Hutchinson Cancer Research Center, Seattle, WA, USA; Public Health Sciences Division, Fred Hutchinson Cancer Research Center, Seattle, WA, USA; Division of Cancer Epidemiology and Genetics, National Cancer Institute, National Institutes of Health, Bethesda, MD, USA; Division of Clinical Epidemiology and Aging Research, German Cancer Research Center (DKFZ), Heidelberg, Germany; Department of Preventive Oncology, German Cancer Research Center (DKFZ), National Center for Tumor Diseases (NCT), Heidelberg, Germany; German Cancer Consortium (DKTK), German Cancer Research Center (DKFZ), Heidelberg, Germany; Centre for Epidemiology and Biostatistics, Melbourne School of Population and Global Health, The University of Melbourne, Parkville, Victoria, Australia; Department of Clinical Pathology, Colorectal Oncogenomics Group, The University of Melbourne, Parkville, Victoria, Australia; University of Melbourne Centre for Cancer Research, Victorian Comprehensive Cancer Centre, Parkville, Victoria, Australia; Genomic Medicine and Family Cancer Clinic, Royal Melbourne Hospital, Melbourne, Victoria, Australia; Epidemiology Research Program, American Cancer Society, Atlanta, GA, USA; Division of Public Health Sciences, Department of Surgery, Washington University School of Medicine, St Louis, MO, USA; Alvin J. Siteman Cancer Center at Barnes-Jewish Hospital and Washington University School of Medicine, St. Louis, MO, USA; Division of Gastroenterology, Department of Medicine, Washington University School of Medicine, St. Louis, MO, USA; Division of Gastroenterology, Massachusetts General Hospital, and Harvard Medical School, Boston, MA, USA; Channing Division of Network Medicine, Brigham and Women’s Hospital, Harvard Medical School, Boston, MA, USA; Clinical and Translational Epidemiology Unit, Massachusetts General Hospital, and Harvard Medical School, Boston, MA, USA; Division of Cancer Epidemiology, German Cancer Research Center, Heidelberg, Germany; Genetic Tumour Epidemiology Group, University Cancer Center Hamburg, University Medical Center Hamburg-Eppendorf, Hamburg, Germany; Lunenfeld Tanenbaum Research Institute, Mount Sinai Hospital, University of Toronto, Toronto, Ontario, Canada; Department of Medical Oncology, Dana-Farber Cancer Institute, Boston, MA, USA; Cancer Epidemiology & Intelligence Division, Cancer Council Victoria, Melbourne, Australia; Melbourne School of Population and Global Health, The University of Melbourne, Melbourne, Australia; Nutrition and Metabolism Section, International Agency for Research on Cancer, World Health Organization, Lyon, France; Centre for Epidemiology and Biostatistics, Melbourne School of Population and Global Health, The University of Melbourne, Parkville, Victoria, Australia; Centre for Epidemiology and Biostatistics, Melbourne School of Population and Global Health, The University of Melbourne, Parkville, Victoria, Australia; Public Health Sciences Division, Fred Hutchinson Cancer Research Center, Seattle, WA, USA; Oncology Data Analytics Program, Catalan Institute of Oncology-IDIBELL, L’Hospitalet de Llobregat, Barcelona, Spain; Department of Medical Oncology, Dana-Farber Cancer Institute, Boston, MA, USA; Public Health Sciences Division, Fred Hutchinson Cancer Research Center, Seattle, WA, USA; Department of Epidemiology, University of Washington, Seattle, WA, USA; Department of Pathology, Dana-Farber Cancer Institute and Harvard Medical School, Boston, MA, USA; Department of Epidemiology, Harvard T.H. Chan School of Public Health, Boston, MA, USA; Public Health Sciences Division, Fred Hutchinson Cancer Research Center, Seattle, WA, USA; Department of Epidemiology, University of Washington, Seattle, WA, USA; Public Health Sciences Division, Fred Hutchinson Cancer Research Center, Seattle, WA, USA; Division of Research, Kaiser Permanente Northern California, Oakland, CA, USA; Department of Medicine and Epidemiology, University of Pittsburgh Medical Center, Pittsburgh, PA, USA; Department of Internal Medicine, University of Utah Health Sciences Center, Salt Lake City, UT, USA; Division of Gastroenterology, Massachusetts General Hospital, and Harvard Medical School, Boston, MA, USA; Clinical and Translational Epidemiology Unit, Massachusetts General Hospital, and Harvard Medical School, Boston, MA, USA; Department of Nutrition, Harvard T.H. Chan School of Public Health, Harvard University, Boston, MA, USA; Public Health Sciences Division, Fred Hutchinson Cancer Research Center, Seattle, WA, USA; Division of Laboratory Genetics, Department of Laboratory Medicine and Pathology, Mayo Clinic, Rochester, MN, USA; Departments of Cancer Biology and Genetics and Internal Medicine, Comprehensive Cancer Center, The Ohio State University, Columbus, OH, USA; Department of Radiation Sciences, Oncology Unit, Umeå University, Umeå, Sweden; Discipline of Genetics, Faculty of Medicine, Memorial University of Newfoundland, St. John’s, Newfoundland & Labrador, Canada; Public Health Sciences Division, Fred Hutchinson Cancer Research Center, Seattle, WA, USA; Division of Clinical Epidemiology and Aging Research, German Cancer Research Center (DKFZ), Heidelberg, Germany; Public Health Sciences Division, Fred Hutchinson Cancer Research Center, Seattle, WA, USA; Department of Epidemiology, University of Washington, Seattle, WA, USA

## Abstract

**Background:**

Smoking is associated with colorectal cancer (CRC) risk. Previous studies suggested this association may be restricted to certain molecular subtypes of CRC, but large-scale comprehensive analysis is lacking.

**Methods:**

A total of 9789 CRC cases and 11 231 controls of European ancestry from 11 observational studies were included. We harmonized smoking variables across studies and derived sex study–specific quartiles of pack-years of smoking for analysis. Four somatic colorectal tumor markers were assessed individually and in combination, including *BRAF* mutation, *KRAS* mutation, CpG island methylator phenotype (CIMP), and microsatellite instability (MSI) status. A multinomial logistic regression analysis was used to assess the association between smoking and risk of CRC subtypes by molecular characteristics, adjusting for age, sex, and study. All statistical tests were 2-sided and adjusted for Bonferroni correction.

**Results:**

Heavier smoking was associated with higher risk of CRC overall and stratified by individual markers (*P*_trend_ < .001). The associations differed statistically significantly between all molecular subtypes, which was the most statistically significant for CIMP and *BRAF*. Compared with never-smokers, smokers in the fourth quartile of pack-years had a 90% higher risk of CIMP-positive CRC (odds ratio = 1.90, 95% confidence interval = 1.60 to 2.26) but only 35% higher risk for CIMP-negative CRC (odds ratio = 1.35, 95% confidence interval = 1.22 to 1.49; *P*_difference_ = 2.1 x 10^-6^). The association was also stronger in tumors that were *CIMP* positive, MSI high, or *KRAS* wild type when combined (*P*_difference_ < .001).

**Conclusion:**

Smoking was associated with differential risk of CRC subtypes defined by molecular characteristics. Heavier smokers had particularly higher risk of CRC subtypes that were CIMP positive and MSI high in combination, suggesting that smoking may be involved in the development of colorectal tumors via the serrated pathway.

Colorectal cancer (CRC) is one of the most common and fatal cancers (1). In the United States, there were an estimated 145 600 new cases and 51 020 deaths in 2019 (2). In addition, CRC is a disease with considerable genetic and molecular heterogeneity (3). Molecular classification of CRC using clinically informative genetic and epigenetic features has potential prognostic (4) and treatment implications (5). Mutations in the *KRAS* gene have been shown to promote the growth of colorectal adenomas in 30%-40% of sporadic CRC (6). Microsatellite instability (MSI), characterized by frequent alterations in tandemly repeated DNA sequences, has been reported to occur in 10%-15% of CRC and associated with a favorable prognosis (7,8). In addition, many MSI-high CRC also present the CpG island methylator phenotype (CIMP) or *BRAF* c.1799T>A (p.V600E) mutations (9).

Cigarette smoking has been established as a risk factor for CRC (10,11). Meta-analysis showed that current smokers had a 17% higher risk of developing CRC and 40% higher risk of CRC mortality than never-smokers (11). Recent evidence suggests that the association between smoking, including current smoking status, cumulative pack-years, duration of smoking or cessation periods, and CRC risk may differ by molecular characteristics. Several studies have found that smoking status has stronger associations with higher risks of MSI-high, CIMP-positive, or *BRAF*-mutated colorectal tumors but is less pronounced among MSI-low or microsatellite stable, CIMP-negative, or *BRAF*–wild-type CRC (12–19). In addition, heavier smoking was found to be associated with an increased risk for *KRAS*–wild-type CRC but not *KRAS*-mutated tumors (20,21). However, 2 studies found no statistically significant difference in the association between smoking and CRC risk by *KRAS* mutation (15,22). Recent meta-analyses showed a statistically significant positive correlation between ever-smoking and *BRAF* mutation, MSI high, and CIMP positivity in CRC (23,24).

However, most studies assessed CRC molecular subtypes only by individual marker status. In this study, we aimed to comprehensively assess the association between smoking and CRC risk both by individual markers (MSI status, CIMP status, *KRAS* and *BRAF* mutations) and by combinations of all 4 markers, using pooled individual-level data from a large consortium.

## Methods

### Study Participants

This study consisted of 9789 patients diagnosed with CRC and 11 231 controls from 11 observational studies within the Genetics and Epidemiology of Colorectal Cancer Consortium and the Colon Cancer Family Registry with available tumor marker and smoking data. Participating studies were previously described and summarized in Table 1 (25,26). All participants provided written informed consent, and each study was approved by the relevant research ethics committee or institutional review board.

**Table 1.** Demographic characteristics of participating studies^a^

Study	Country (enrollment year)	Study design	Cases		Controls		Mean (SD) age, y	Female, No. (%)
			No.	% never-smokers	No.	% never-smokers		
CCFR	United States, Canada, Australia (1996-2015)	Case-control	2636	45.5	2083	47.2	54.0 (11.7)	2477 (52.5)
CPSII	United States (1992-1999)	Cohort	858	40.2	969	45.6	74.3 (6.6)	912 (49.9)
DACHS	Germany (2003-2016)	Case-control	2322	46.7	3428	50.0	68.7 (10.6)	2291 (39.8)
DALS	United States (1990-1993)	Case-control	1096	42.7	1163	49.4	65.4 (9.7)	1017 (45.0)
EDRN	United States (2012-2013)	Case-control	195	56.9	349	73.1	60.5 (11.4)	262 (48.2)
EPIC	Sweden (1992-1998)	Case-control	115	49.6	318	54.1	67.2 (7.7)	206 (47.6)
HPFS	United States (1986-2012)	Cohort	584	37.5	433	46.2	71.0 (8.9)	0 (0)
MCCS	Australia (1990-1994)	Cohort	490	51.2	674	53.0	68.9 (8.8)	553 (47.5)
NFCCR	Newfoundland (2000-2004)	Case-control	477	28.3	458	37.6	59.9 (9.1)	376 (40.2)
NHS	United States (1976-2013)	Cohort	783	40.0	1071	44.1	67.7 (8.3)	1854 (100)
NSHDS	Sweden (1995-2005)	Case-control	233	38.2	285	45.6	62.6 (8.2)	250 (48.3)
Total			9789	43.6	11 231	48.7	64.8 (11.9)	10 198 (48.5)

a CCFR = Colorectal Cancer Family Registry; CPSII = Cancer Society Cancer Prevention Study II; DACHS = Darmkrebs: Chancen der Verhütung durch Screening Study; DALS = Diet, Activity and Lifestyle Study; HPFS = Health Professionals Follow-up Study; Kentucky = the Kentucky case-control study; MCCS = Melbourne Case-Control Study (in Melbourne Collaborative Cohort); NFCCR = NewFoundland Case-Control Study; NHS = Nurses’ Health Study; NSHDS = the Northern Sweden Health and Disease Study.

CRC cases were confirmed by medical record, pathology report, or death certificate by study protocol. Controls were individuals without history of CRC at the time of case selection and were selected per study-specific matching criteria. Participants of non-European ancestry were excluded from the analysis because of small sample size.

### Assessment and Harmonization of Tumor Marker Data

Details on data collection and harmonization of tumor marker data were summarized and published previously (25–27). Briefly, testing for MSI, *BRAF* gene mutations, *KRAS* gene mutations, and CIMP status was conducted previously by each study and according to individual study protocols. To harmonize markers across all studies, we created 2 categories for each marker for downstream analyses. In instances where studies categorized as MSI high (MSI-H), MSI low (MSI-L), and microsatellite stable (MSS), we collapsed MSI-L and MSS into an MSI-L/MSS category. In instances where studies categorized as CIMP high, CIMP low, and CIMP negative, we collapsed CIMP-low and CIMP-high into the CIMP-positive (CIMP+) category. We included any mutation identified by a study for *BRAF* and *KRAS* genes.

**Figure 1.**
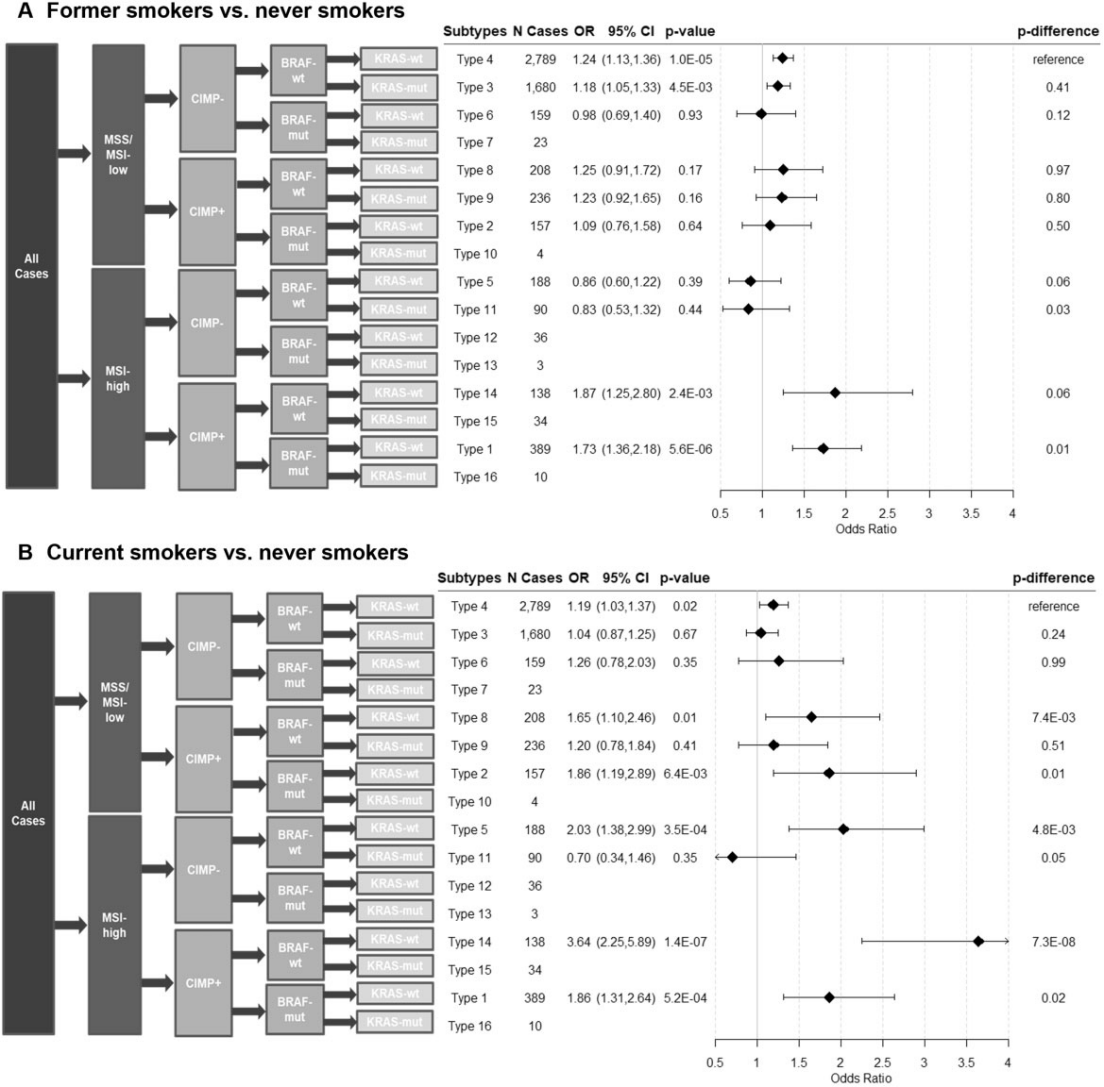
Associations between former and current smokers and risk of CRC subtypes defined by combined marker status. Two-sided Wald test was used to calculate the *P* values from the case-control analysis (N controls = 11 231) and case-only analysis (*P*_difference_). A Bonferroni corrected *P* value threshold of 5.0 x 10^-3^ was used for both case-control and case-only analyses. Error bars represent the 95% confidence intervals (CIs). CIMP = CpG island methylation phenotype; CRC = colorectal cancer; MSI = microsatellite instability; MSS = microsatellite stable; mut = mutated; OR = odds ratio; wt = wild type.

Additionally, we combined markers to create subtype classifications: subtypes 1-5 were created according to JASS classification (28), and type 6-16 were numbered consecutively by the status of MSI, CIMP, *BRAF*, and *KRAS* (summarized in Figure 1). Only cases with all markers assessed are included in combined molecular classifications and corresponding analysis.

### Smoking and Other Exposure Data

Data collection and harmonization of epidemiologic data have been described elsewhere (29,30). Briefly, demographic and environmental risk factors were self-reported at in-person interviews or via structured self-administered questionnaires. Data were collected at study entry, blood draw, or 1 to 2 years prior to sample ascertainment. A multistep, iterative data harmonization procedure was applied, and multiple quality-control checks were performed, reconciling each study’s unique protocols. Variables were combined into a single dataset with common definitions, standardized coding, and standardized permissible values.

Smoking status was categorized into never-, former, and current smokers at baseline in each study. In addition, sex study–specific quartiles were created for smoking pack-years among ever-smokers. Never-smokers were used as a reference group in all analyses.

Demographic variables included age and sex. Age was defined as age at diagnosis for cases and age at selection for controls. Other lifestyle covariates included body mass index (BMI; defined as weight[kg]/height[cm^2^]), regular use of non-steroidal anti-inflammatory drugs (NSAIDs), history of colorectal screening, alcohol intake, and physical activity.

### Statistical Analyses

All statistical tests were 2-sided. The distributions of individual tumor markers were summarized among CRC cases, and Pearson correlation test was used to assess the correlation among markers. We used multinomial logistic regression models to estimate odds ratios (OR) and 95% confidence intervals (CIs) for the association of smoking with the risk of CRC subtypes. To account for multiple testing in case-control analysis, we used a Bonferroni corrected *P* value threshold of .05/16 (4 markers x 2 status x 2 smoking comparisons) = 3.1 x 10^-3^ for categorical smoking status and .05/8 (4 markers x 2 status x 1 group linear comparison) = .006 for smoking pack-years. We used logistic regression models to assess the differences in the associations between smoking and molecularly mutated subtypes (*BRAF* mutated [mut], *KRAS*-mut, MSI-H, or CIMP+), as compared with wild-type subtypes (*BRAF*-wild type [wt], *KRAS*-wt, MSI-L/MSS, or CIMP negative [CIMP-], respectively) among cases only (Bonferroni corrected *P*_difference_ threshold: .05/8 = 6.3 x 10^-3^ for categorical smoking status and .05/4 = 0.013 for smoking pack-years). Age at diagnosis, sex, and study were adjusted as covariates in the models. According to a priori knowledge about CRC risk factors that have been associated with smoking, we further simultaneously adjusted for BMI, use of NSAIDs, history of screening, alcohol intake, and physical activity as sensitivity analyses.

In analysis of combined marker status, CRC subtypes with at least 50 cases were assessed in their association with smoking. Similarly, we used multinomial logistic regression models, adjusting for age, sex, and study. The subtype with MSI-L/MSS, CIMP-, *BRAF*-wt and *KRAS*-wt was used as a reference group in the case-only analysis (Bonferroni corrected *P*_difference_ threshold: .05/10 = 5.0 x 10^-3^).

Exploratory analysis of smoking–CRC association stratified by sex, colonic locations, and study design was also conducted for both individual and combined markers. All analyses were performed using R version 3.5.1 .

## Results

### Overall Distributions of Tumor Markers

Among the 21 020 participants, there was a larger proportion of never-smokers among controls (48.7%) than among CRC cases (43.6%; Table 1). Among the 9789 CRC cases with tumor data available, 11% of the tumors were *BRAF*-mut, 34% *KRAS*-mut, 15% MSI-H, and 18% CIMP+ (Table 2). In addition, the MSI-H, CIMP+, and *BRAF*-mut subtypes were highly positively correlated with each other (Pearson correlation > 0.4; frequency presented in Supplementary Table 1, available online). *KRAS*-mut tumors were inversely correlated with other markers (Pearson correlation < −0.1).

**Table 2.** Association between former or current smoking and individual molecular subtypes of colorectal cancer, compared with never-smokers

Marker and status	No. of cases	Never-smokers	Smokers					
			Former smokers			Current smokers		
		OR (95% CI)	OR (95% CI)	*P* ^a^	*P*_difference_ ^b^	OR (95% CI)	*P* ^a^	*P*_difference_ ^b^
BRAF								
mut	1008	1.00 (Referent)	1.43 (1.23 to 1.66)	3.9 x 10^-6^		1.89 (1.54 to 2.33)	1.5 x 10^-9^	
wt	7695	1.00 (Referent)	1.17 (1.09 to 1.25)	7.5 x 10^-6^	.03	1.28 (1.16 to 1.41)	1.7 x 10^-6^	1.0 x 10^-4^
KRAS								
mut	2484	1.00 (Referent)	1.16 (1.05 to 1.28)	3.6 x 10^-3^		1.11 (0.95 to 1.29)	.18	
wt	4892	1.00 (Referent)	1.21 (1.12 to 1.31)	1.8 x 10^-6^	.36	1.40 (1.25 to 1.56)	5.0 x 10^-9^	3.3 x 10^-3^
CIMP								
+	1382	1.00 (Referent)	1.37 (1.20 to 1.56)	2.1 x 10^-6^		1.82 (1.52 to 2.18)	8.6 x 10^-11^	
−	6232	1.00 (Referent)	1.16 (1.08 to 1.25)	5.2 x 10^-5^	.03	1.22 (1.09 to 1.36)	3.7 x 10^-4^	5.6 x 10^-7^
MSI								
MSI-H	1334	1.00 (Referent)	1.27 (1.11 to 1.46)	5.4 x 10^-4^		2.01 (1.68 to 2.40)	1.1 x 10^-14^	
MSI-L/MSS	7828	1.00 (Referent)	1.18 (1.10 to 1.26)	2.1 x 10^-6^	.43	1.27 (1.15 to 1.40)	3.8 x 10^-6^	1.2 x 10^-6^

a Two-sided Wald test was used to calculate the *P* values from the case-control analysis (N controls = 11 231). A Bonferroni corrected *P* value threshold of 6.3 x 10^-3^ was used for case-control analyses. CI = confidence interval; CIMP = CpG island methylation phenotype; MSI = microsatellite instability; MSI-H = MSI-high; MSI-L = MSI-low; MSS = microsatellite stable; mut = mutated; OR = odds ratio; wt = wild type.

b Two-sided Wald test was used to calculate the *P* values from the case-only analysis. A Bonferroni corrected *P* value threshold of 3.1 x 10^-3^ was used for case-only analyses.

### Association Between Smoking and Individual Marker Subtypes

In case-control analysis, individuals who smoked were associated with a higher risk of CRC overall and stratified by individual marker subtypes except for *KRAS*-mut tumors (*P* < 3.1 x 10^-3^; Table 2). Associations were stronger among current smokers. For instance, current smoking was associated with a 2-fold risk in MSI-H CRC (OR = 2.01, 95% CI = 1.68 to 2.40), whereas former smoking was associated with higher risk in MSI-H CRC (OR = 1.27, 95% CI = 1.11 to 1.46), compared with never-smokers. In case-only analyses, the association between smoking status and CRC risk was statistically significantly stronger for *BRAF*-mut, *KRAS*-wt, MSI-H, and CIMP+ CRC subtypes among current smokers only but not among former smokers, after Bonferroni correction (*P*_difference_ < 6.3 x 10^-3^; Table 2).

We further assessed the dose-response relationship between smoking and CRC subtypes. Compared with nonsmokers, higher pack-years of smoking were associated with higher risk of CRC among all subtypes in case-control analysis (*P* < 1.6 x 10^-3^; *P*_trend_ < .001; Table 3). In case-only analysis, the association between pack-years and molecular subtypes was statistically significantly stronger for *BRAF*-mut, CIMP+, and MSI-H subtypes compared with wild-type or negative CRC cases after Bonferroni correction (*P*_difference_ < 6.3 x 10^-3^; Table 3). The largest difference in case-control risk estimates were seen for *BRAF*-mut and CIMP+ CRC. Participants in the highest quartile of smoking pack-years had nearly a 2-fold risk for CRC if they had *BRAF*-mut (OR = 1.92, 95% CI = 1.58 to 2.33) or CIMP+ tumors (OR = 1.90, 95% CI = 1.60 to 2.26), compared with nonsmokers, respectively. In comparison, the risk of *BRAF*-wt or CIMP- CRC was increased by only 36.9% (*P*_difference_ = 2.7 x 10^-6^) and 34.8% (*P*_difference_ = 2.4 x 10^-6^) among heaviest smokers, respectively. There was no statistically significant difference after Bonferroni correction in the associations of pack-years of smoking with *KRAS*-mut or *KRAS*-wt CRC (*P*_difference_ = 9.6 x 10^-3^). Sensitivity analysis that additionally adjusted for BMI, family history of CRC, CRC screening history, NSAID use, or alcohol intake did not meaningfully change our conclusions (data not shown).

**Table 3.** Association between quartiles of smoking pack-years and individual molecular subtypes of colorectal cancer compared with nonsmokers

Marker and status	No. of cases	Smoking, pack-years				*P*_trend_ ^a^	*P*_difference_ ^b^
		Quartile 1 OR (95% CI)	Quartile 2 OR (95% CI)	Quartile 3 OR (95% CI)	Quartile 4 OR (95% CI)		
BRAF							
mut	921	0.81 (0.62 to 1.06)	1.49 (1.20 to 1.84)	1.69 (1.37 to 2.08)	1.92 (1.58 to 2.33)	1.0 x 10^-14^	
wt	7233	0.99 (0.89 to 1.11)	1.21 (1.09 to 1.34)	1.16 (1.05 to 1.29)	1.37 (1.24 to 1.51)	2.5 x 10^-11^	2.7 x 10^-6^
KRAS							
mut	2331	1.04 (0.89 to 1.21)	1.12 (0.96 to 1.29)	1.17 (1.01 to 1.35)	1.23 (1.07 to 1.41)	1.0 x 10^-3^	
Wt	4547	0.93 (0.82 to 1.06)	1.28 (1.14 to 1.43)	1.22 (1.09 to 1.37)	1.48 (1.33 to 1.65)	2.1 x 10^-14^	9.6 x 10^-3^
CIMP							
+	1247	1.05 (0.85 to 1.30)	1.33 (1.09 to 1.62)	1.62 (1.34 to 1.95)	1.90 (1.60 to 2.26)	8.9 x 10^-16^	
−	5966	1.00 (0.89 to 1.12)	1.20 (1.08 to 1.33)	1.15 (1.03 to 1.28)	1.35 (1.22 to 1.49)	2.1 x 10^-9^	2.4 x 10^-6^
MSI							
MSI-H	1239	1.10 (0.90 to 1.35)	1.26 (1.04 to 1.53)	1.53 (1.28 to 1.84)	1.66 (1.39 to 1.99)	9.0 x 10^-11^	
MSI-L/MSS	7255	0.98 (0.89 to 1.09)	1.24 (1.12 to 1.36)	1.16 (1.05 to 1.29)	1.38 (1.25 to 1.52)	7.0 x 10^-12^	3.9 x 10^-3^

a Two-sided Wald test was used to calculate the *P* values from the case-control analysis (N controls = 10 199). A Bonferroni corrected *P* value threshold of 6.3 x 10^-3^ was used for case-control analyses. CI = confidence interval; CIMP = CpG island methylation phenotype; MSI = microsatellite instability; MSI-H = MSI-high; MSI-L = MSI-low; MSS = microsatellite stable; mut = mutated; OR = odds ratio; wt = wild type.

b Two-sided Wald test was used to calculate the *P* values from the case-only analysis. A Bonferroni corrected *P* value threshold of .013 for case-only analyses.

### Association Between Smoking and Combined Marker Subtypes

Overall distribution of CRC cases by smoking status and combined marker subtypes are summarized in Supplementary Figure 1 (available online). Of 16 possible combined CRC subtypes, 10 had 50 or more cases and were included in the analysis. Among them, former smoking was statistically significantly associated with higher risk of 4 CRC subtypes after Bonferroni correction (types 4, 3, 14, 1; Figure 1, A) compared with never-smokers. Comparatively, current smoking was associated with higher risk of CRC for 6 subtypes, but only 3 remained statistically significant after Bonferroni correction (types 5, 14, 1; Figure 1, B). The strongest association for both former and current smoking was observed in type 14 (MSI-H, CIMP+, *BRAF*-wt, and *KRAS*-wt), where former and current smoking was associated with 87% and 264% higher risk of type 14 CRC compared with never-smokers, respectively. Using type 4 (all markers wild type/negative) as reference in case-only analyses, we observed no statistically significant differences between risks of CRC subtypes among former smokers. However, cases with current smoking were statistically significantly more likely to be type 5 (only MSI-H; *P*_difference_ = 4.8 x 10^-3^) and type 14 CRC (*P*_difference_ = 7.3 x 10^-8^) compared with never-smokers after Bonferroni correction (Figure 1, B).

**Figure 2.**
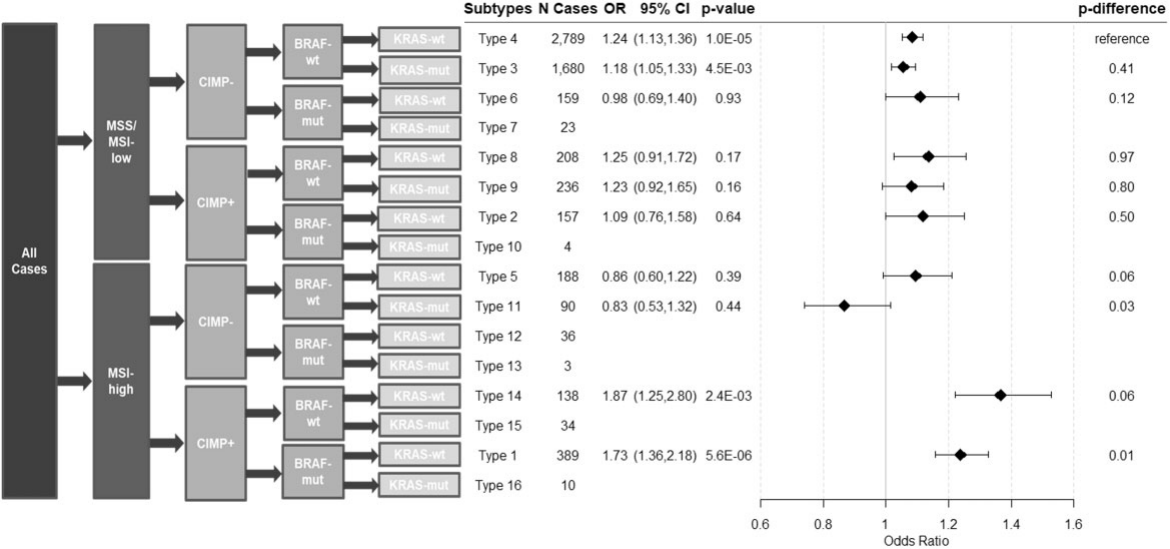
Associations between smoking pack-years and risk of CRC subtypes defined by combined marker status. Two-sided Wald test was used to calculate the *P* values from the case-control analysis (N control = 10 199) and case-only analysis (*P*_difference_). A Bonferroni corrected *P* value threshold of .005 was used for both case-control and case-only analyses. Error bars represent the 95% confidence intervals (CIs). CIMP = CpG island methylation phenotype; CRC = colorectal cancer; MSI = microsatellite instability; MSS = microsatellite stable; mut = mutated; OR = odds ratio; wt = wild type.

Higher smoking pack-years was also statistically significantly associated with higher risk of 4 CRC subtypes after Bonferroni correction (types 4, 3, 14, and 1; Figure 2). Similarly, the association between smoking pack-years and CRC risk was strongest in type 14 (OR per quartile = 1.37, 95% CI = 1.22 to 1.53). When compared with type 4 in case-only analyses, higher smoking pack-years was associated only with higher risk of type 14 (*P*_difference_ = 1.8 x 10^-5^) and type 1 CRC (MSI-H, CIMP+, *BRAF*-mut, and *KRAS*-wt; *P*_difference_ = 2.5 x 10^-4^).

### Exploratory Stratified Analysis

When stratified by colonic location, *BRAF*-mut, CIMP+, and MSI-H status were more frequent in proximal colon cancer, compared with distal colon or rectal cancer (Supplementary Table 2, available online). For proximal colon, current smoking and higher pack-years were associated with higher risk of *BRAF*-mut, *KRAS*-wt, CIMP+, and MSI-H tumors (Supplementary Tables 3 and 4, available online) and higher risk of type 14 (Supplementary Figures 2 and 3, available online). Although sample sizes were limited, a similar trend of smoking–CRC association was observed in distal colon and rectal cancer. Current smoking was associated with higher risk of type 8 distal colon cancer (only CIMP+), but this did not remain statistically significant after Bonferroni correction (Supplementary Figure 2, available online). Interaction analysis between smoking and colonic location was not statistically significant.

When stratified by sex, similar trends of associations were observed, although the risk estimates varied slightly between sexes. For instance, current smoking was associated with a 90% and 66% increase in CIMP+ tumors among females and males, respectively (Supplementary Tables 5 and 6, available online). In addition, the dose–response association was stronger for *BRAF*-mut and CIMP+ CRC among females. In combined marker analysis, current smoking was most strongly associated with type 14 CRC in both sexes (Supplementary Figures 4 and 5, available online). Interaction analysis between smoking and sex was not statistically significant. Further stratification by sex in proximal colon tumors did not suggest statistically significant difference (data not shown). In addition, similar trends were also observed when stratified by study design (Supplementary Tables 7 and 8 and Supplementary Figures 6 and 7, available online).

## Discussion

In this large study, we found that smoking was associated with higher risk of all molecular CRC subtypes, and the association was statistically significantly stronger for *BRAF*-mut, MSI-H, or CIMP+ CRC cases. We also found that smoking had a statistically significantly stronger association with CRC subtypes that display MSI-H and CIMP+ status.

Our results are consistent with previous evidence that smoking is associated with higher risk of CRC subgroups classified by individual marker status, including similar findings from 1 of the participating studies (19). Current smoking was associated with almost 2-fold higher risk of CRC with MSI-H, CIMP+, or *BRAF*-mut compared with never smoking (12). A study in 2 prospective cohorts found that a longer cessation period was associated with MSI-H and CIMP+ CRC, but not with MSS or CIMP- CRC, compared with current smokers (13). In addition, longer duration of smoking was found to be associated with increased risk of MSI-H CRC (14). Several cohort and case-control studies also found that higher smoking pack-years were associated with higher risks of CRC with MSI-H, CIMP+, or *BRAF*-mut, compared with wild-type or negative CRC subtypes (12–14,17). In a population-based, case-control study, current cigarette smoking and higher pack-years were found to be statistically significantly associated with higher risk of MSI-H than MSS colon tumors (16). Similar to our results on *KRAS* mutation status, several observational studies found that smoking status and pack-years were associated only with higher risk of *KRA*S-wt but not *KRAS*-mut tumors, although the differences were not statistically significant (6,20,21). In contrast, a case-cohort study (648 cases) in the Netherlands observed a non–statistically significant increase in *KRAS*-wt CRC risk among former smokers but not among current smokers (20).

Individual markers were not independent from each other. CIMP+ CRC tumors tend to have a high frequency of MSI and *BRAF* mutation (9,31–34). However, few studies have assessed the combined subtypes of CRC. A prospective cohort study found that smoking 20 or more cigarettes per day was associated with higher risks of MSI-L/MSS and CIMP+ CRC, regardless of *BRAF* mutation status (17). No statistically significant association was found in CIMP- tumors. Another analysis in 2 prospective cohorts also found that smoking 40 or more pack-years of cigarettes was associated with higher risk of CIMP+ and MSI-H CRC compared with never-smokers (13). Consistent with previous findings, we found that higher smoking pack-years were statistically significantly associated with higher risk of CIMP+ and MSI-H CRC, regardless of *BRAF* mutation status.

Smoking is a well-established carcinogen for CRC (35). Meta-analyses of epidemiological studies have consistently found a statistically significant association, and dose-response relationships, between smoking and CRC risk (10,11,36). However, knowledge on the underlying mechanisms of smoking in CRC molecular subtypes is limited. In general, tobacco smoke contains a variety of toxic chemicals (37), many of which can induce DNA damage (38). Tobacco exposure has also been associated with CIMP in other cancer types, including lung (39,40), bladder (41), and head and neck cancer (42). Therefore, it is biologically plausible that smoking promotes colorectal tumor growth and progression by epigenetic alterations. In addition, the detoxification of smoking-induced carcinogens are metabolized by phase I and phase II enzymes such as *CYP* (P-450) family genes, which lead to the production of abnormal DNA and mutations in genes such as *KRAS* and *BRAF* (43).

Furthermore, the CIMP+ and MSI-H tumors are more likely to arise from serrated polyp pathways, as compared with traditional adenoma-carcinoma pathways. It is estimated that 10%-20% of CRCs arise via serrated polyp-carcinoma pathway (44). DNA methylation is key to the development of this type of cancer (45). CIMP+ phenotype is frequently observed in precursor serrated lesions and colorectal polyps, ranging between 40% and 80% (46,47). MSI-H phenotype has also been observed in 20%-36% of serrated adenomas (48,49). Consistent with previous evidence, our exploratory analysis showed that *BRAF*-mut, CIMP+, and MSI-H were preferentially located in the proximal colon. However, we observed similar associations with small variations, when stratified by location, suggesting that our finding cannot be explained by tumor location. It is also estimated that serrated adenocarcinoma has a less favorable survival than traditional adenocarcinoma (50), which could be partially because of the interaction between smoking and the enrichment of *BRAF* mutations and CIMP expression levels. Therefore, better understanding of the risk factors of these molecular characteristics may help provide insights to the trajectory of serrated carcinogenesis and preventive and therapeutic implications.

Several features in our study provided the opportunity to systematically evaluate associations between smoking and molecular subgroups of CRC. First, this is the largest study to investigate these associations with sufficient statistical power for primary analysis. In addition, we combined CRC subtypes by all 4 tumor markers, providing a more comprehensive analysis for tumor characteristics. With sufficient sample size, we were the first to extend the combined subtype analysis beyond 5 previously defined subtypes (28) and thus found a statistically significant association between smoking and new subtypes, suggesting a stronger impact of smoking on the serrated polyp-carcinoma pathway. Furthermore, smoking variables and other CRC risk factors were assessed and harmonized among all participating studies, which allowed us to further adjust for potential confounders in sensitivity analysis.

There are also limitations. We did not investigate all 16 possible combinations of CRC subtypes, and the conclusions could not be inferred for the rarer subtypes. Both case-control and cohort studies were included. There is a possibility of misclassification of smoking status, especially in case-control studies because of recall bias. However, we observe similar trends of smoking-CRC associations when stratified by study design We also found almost identical estimates using random-effect meta-analysis across study-specific estimates of smoking–CRC associations (data not shown). In exploratory stratified analysis, we found potential variation in smoking–CRC association by sex or colonic locations. However, these exploratory results warrant further investigation in the future. Although we adjusted for several potential confounders in sensitivity analysis, we could not rule out the possibility of unmeasured confounding. Lastly, our study population was of European ancestry only. Therefore, our conclusions may not be generalizable to other race and ethnicity groups.

In conclusion, we found that heavier smoking was associated with higher risk among all subtypes of CRC, particularly for those that may arise from serrated polyp pathways. These findings may help better understand the tumorigenesis of serrated adenomas and provide insights to targeted CRC prevention and treatment.

## Funding

Genetics and Epidemiology of Colorectal Cancer Consortium (GECCO): National Cancer Institute (NCI), National Institutes of Health (NIH), US Department of Health and Human Services (U01 CA164930, U01 CA137088, R01 CA059045, U01 CA164930). Genotyping/Sequencing services were provided by the Center for Inherited Disease Research (CIDR) (X01-HG008596 and X-01-HG007585). CIDR is fully funded through a federal contract from the National Institutes of Health to Johns Hopkins University, contract number HHSN268201200008I. This research was funded in part through the NIH/NCI Cancer Center Support Grant P30 CA015704. The Colon Cancer Family Registry (CCFR, www.coloncfr.org) is supported in part by funding from the NCI, NIH (award U01 CA167551). The CCFR Set-1 (Illumina 1 M/1M-Duo) and Set-2 (Illumina Omni1-Quad) scans were supported by NIH awards U01 CA122839 and R01 CA143247 (to GC). The CCFR Set-3 (Affymetrix Axiom CORECT Set array) was supported by NIH award U19 CA148107 and R01 CA81488 (to SBG). The CCFR Set-4 (Illumina OncoArray 600 K SNP array) was supported by NIH award U19 CA148107 (to SBG) and by the CIDR, which is funded by the NIH to Johns Hopkins University, contract number HHSN268201200008I. The SCCFR Illumina HumanCytoSNP array was supported through NCI award R01 CA076366 (to PAN). Additional funding for the OFCCR/ARCTIC was through award GL201-043 from the Ontario Research Fund (to BWZ), award 112746 from the Canadian Institutes of Health Research (to TJH), through a Cancer Risk Evaluation (CaRE) Program grant from the Canadian Cancer Society (to SG) and through generous support from the Ontario Ministry of Research and Innovation. The content of this manuscript does not necessarily reflect the views or policies of the NCI, NIH, or any of the collaborating centers in the CCFR, nor does mention of trade names, commercial products, or organizations imply endorsement by the US Government, any cancer registry, or the CCFR. CPS-II: The American Cancer Society funds the creation, maintenance, and updating of the Cancer Prevention Study-II (CPS-II) cohort. This study was conducted with institutional review board approval. DACHS: This work was supported by the German Research Council (BR 1704/6-1, BR 1704/6-3, BR 1704/6-4, CH 117/1-1, HO 5117/2-1, HE 5998/2-1, KL 2354/3-1, RO 2270/8-1 and BR 1704/17-1), the Interdisciplinary Research Program of the National Center for Tumor Diseases (NCT), Germany, and the German Federal Ministry of Education and Research (01KH0404, 01ER0814, 01ER0815, 01ER1505A and 01ER1505B). DALS: National Institutes of Health (R01 CA48998 to ML Slattery). EDRN: This work is funded and supported by the NCI, EDRN Grant (U01 CA 84968-06). EPIC: The coordination of EPIC is financially supported by the European Commission (DGSANCO) and the International Agency for Research on Cancer. The national cohorts are supported by Danish Cancer Society (Denmark); Ligue Contre le Cancer, Institut Gustave Roussy, Mutuelle Générale de l’Education Nationale, Institut National de la Santé et de la Recherche Médicale (INSERM) (France); German Cancer Aid, German Cancer Research Center (DKFZ), Federal Ministry of Education and Research (BMBF), Deutsche Krebshilfe, Deutsches Krebsforschungszentrum and Federal Ministry of Education and Research (Germany); the Hellenic Health Foundation (Greece); Associazione Italiana per la Ricerca sul Cancro-AIRCItaly and National Research Council (Italy); Dutch Ministry of Public Health, Welfare and Sports (VWS), Netherlands Cancer Registry (NKR), LK Research Funds, Dutch Prevention Funds, Dutch ZON (Zorg Onderzoek Nederland), World Cancer Research Fund (WCRF), Statistics Netherlands (the Netherlands); ERC-2009-AdG 232997 and Nordforsk, Nordic Centre of Excellence programme on Food, Nutrition and Health (Norway); Health Research Fund (FIS), PI13/00061 to Granada, PI13/01162 to EPIC-Murcia, Regional Governments of Andalucía, Asturias, Basque Country, Murcia and Navarra, ISCIII RETIC (RD06/0020) (Spain); Swedish Cancer Society, Swedish Research Council and County Councils of Skåne and Västerbotten (Sweden); Cancer Research UK (14136 to EPIC-Norfolk; C570/A16491 and C8221/A19170 to EPIC-Oxford), Medical Research Council (1000143 to EPIC-Norfolk, MR/M012190/1 to EPICOxford) (United Kingdom). Harvard cohorts (HPFS, NHS): HPFS is supported by the National Institutes of Health (P01 CA055075, UM1 CA167552, U01 CA167552, R01 CA137178, R01 CA151993, R35 CA197735, K07 CA190673, and P50 CA127003) and NHS by the National Institutes of Health (R01 CA137178, P01 CA087969, UM1 CA186107, R01 CA151993, R35 CA197735, K07CA190673, and P50 CA127003). MCCS cohort recruitment was funded by VicHealth and Cancer Council Victoria. The MCCS was further supported by Australian NHMRC grants 509348, 209057, 251553 and 504711 and by infrastructure provided by Cancer Council Victoria. Cases and their vital status were ascertained through the Victorian Cancer Registry (VCR) and the Australian Institute of Health and Welfare (AIHW), including the National Death Index and the Australian Cancer Database. NFCCR: This work was supported by an Interdisciplinary Health Research Team award from the Canadian Institutes of Health Research (CRT 43821); the National Institutes of Health, US Department of Health and Human Serivces (U01 CA74783); and National Cancer Institute of Canada grants (18223 and 18226). The authors wish to acknowledge the contribution of Alexandre Belisle and the genotyping team of the McGill University and Génome Québec Innovation Centre, Montréal, Canada, for genotyping the Sequenom panel in the NFCCR samples. Funding was provided to Michael O. Woods by the Canadian Cancer Society Research Institute. NSHDS: Swedish Cancer Society; Cancer Research Foundation in Northern Sweden; Swedish Research Council; J C Kempe Memorial Fund; Faculty of Medicine, Umeå University, Umeå, Sweden; and Cutting-Edge Research Grant from the County Council of Västerbotten, Sweden. OFCCR: The Ontario Familial Colorectal Cancer Registry was supported in part by the NCI/NIH under award U01 CA167551 and award U01/U24 CA074783 (to SG). Additional funding for the OFCCR and ARCTIC testing and genetic analysis was through and a Canadian Cancer Society CaRE (Cancer Risk Evaluation) program grant and Ontario Research Fund award GL201-043 (to BWZ), through the Canadian Institutes of Health Research award 112746 (to TJH), and through generous support from the Ontario Ministry of Research and Innovation. OSUMC: OCCPI funding was provided by Pelotonia and HNPCC funding was provided by the NCI (CA16058 and CA67941). SCCFR: The Seattle Colon Cancer Family Registry was supported in part by the NCI/NIH under awards U01 CA167551, U01 CA074794 (to JDP), and awards U24 CA074794 and R01 CA076366 (to PAN).

## Footnotes

**Role of Funders:** The funders had no role in the design of the study; the collection, analysis, and interpretation of the data; the writing of the manuscript; and the decision to submit the manuscript for publication.

**Disclosures:** X. Wang reports employment (post the completion of this work) at Flatiron Health, Inc, which is an independent subsidiary of the Roche group and stock ownership in Roche. The other authors have no disclosures.

**Author Contributions:** Conception and design: XW, EA, TAH, MH, UP. Development of methodology: XW, EA, TAH, AIP, WS, MH, UP. Acquisition of data (provided animals, acquired and managed patients, provided facilities, etc.): TAH, BLB, SIB, HB, DDB, PTC, YC, ATC, JCC, SJG, MG, GGG, MJG, JLH, MAJ, YL, VM, RN, PAN, SO, AIP, LCS, RES, MLS, MS, WS, SNT, AET, BVG, MOW, LH, MH, UP. Analysis and interpretation of data (eg, statistical analysis, biostatistics, computational analysis): XW, TAH, YL, LH, UP. Writing, review, and/or revision of the manuscript: XW, EA, TAH, BLB, SIB, HB, DDB, PPTC, YC, ATC, JCC, SJG, MG, GGG, MJG, JLH, MAJ, YL, VM, RN, PAN, SO, AIP, LCS, RES, MLS, MS, WS, SNT, AET, BVG, MOW, LH, MH, UP. Administrative, technical, or material support (ie, reporting or organizing data, constructing databases): XW, TAH, YL,. Study supervision: MH, LH, UP.

**Acknowledgements**: CPS-II: The authors thank the CPS-II participants and Study Management Group for their invaluable contributions to this research. The authors would also like to acknowledge the contribution to this study from central cancer registries supported through the Centers for Disease Control and Prevention National Program of Cancer Registries and cancer registries supported by the National Cancer Institute Surveillance Epidemiology and End Results program. DACHS: We thank all participants and cooperating clinicians and everyone who provided excellent technical assistance. EDRN: We acknowledge all the following contributors to the development of the resource: University of Pittsburgh School of Medicine, Department of Gastroenterology, Hepatology and Nutrition and Biomedical Informatics. EPIC: Where authors are identified as personnel of the International Agency for Research on Cancer/World Health Organization, the authors alone are responsible for the views expressed in this article and they do not necessarily represent the decisions, policy, or views of the International Agency for Research on Cancer/World Health Organization. Harvard cohorts (HPFS, NHS): The study protocol was approved by the institutional review boards of the Brigham and Women’s Hospital and Harvard T.H. Chan School of Public Health, and those of participating registries as required. We would like to thank the participants and staff of the HPFS and NHS for their valuable contributions as well as the following state cancer registries for their help: AL, AZ, AR, CA, CO, CT, DE, FL, GA, ID, IL, IN, IA, KY, LA, ME, MD, MA, MI, NE, NH, NJ, NY, NC, ND, OH, OK, OR, PA, RI, SC, TN, TX, VA, WA, WY. The authors assume full responsibility for analyses and interpretation of these data. NSHDS investigators thank the Biobank Research Unit at Umeå University, the Västerbotten Intervention Programme, the Northern Sweden MONICA study, and Region Västerbotten for providing data and samples and acknowledge the contribution from Biobank Sweden, supported by the Swedish Research Council (VR 2017-00650).

## Data Availability

Tumor marker and epidemiologic data is available upon request and permission. Please contact gecco@fredhutch.org to request the standardized proposal form. The principal investigators of each contributing study will evaluate and approve the proposal, and data access will be managed centrally .

## Supplementary Material

pkab056_Supplementary_Data
