# Supplementary material for: Association Between Smoking and Molecular Subtypes of Colorectal Cancer
Source: JNCI Cancer Spectr. 2021 Jun 14;5(4):pkab056. doi: 10.1093/jncics/pkab056 (PMC8346704; doi:10.1093/jncics/pkab056)
Supplement: pkab056_Supplementary_Data [file pkab056_supplementary_data.pdf]

Supplementary Materials

Supplementary Table 1. Relationships between individual marker status among colorectal cancer cases[a]

Markers	Within each subset of colorectal cancer cases, No (%)			
	MSI-high (N=1,334)	CIMP+ (N=1,382)	*BRAF*-mutated (N=1,008)	*KRAS*-mutated (N=2,484)
MSI-high	--	635 (45.9)	536 (53.2)	158 (6.4)
CIMP+	635 (47.6)	--	606 (60.1)	297 (12.0)
BRAF-mutated	536 (40.2)	606 (43.8)	--	46 (1.9)
KRAS-mutated	158 (11.8)	297 (21.5)	46 (4.6)	--

[a] MSI: microsatellite instability; CIMP: CpG island methylation phenotype.

Supplementary Table 2. Sample size and distribution of tumor marker status by colonic location

Marker	Status	Colonic location, No. (%)		
		Proximal colon	Distal colon	Rectum
BRAF	mut	827 (22.5)	157 (5.4)	91 (3.4)
	wt	2,848 (87.5)	2,741 (94.6)	2,596 (96.6)
KRAS	mut	1,222 (37.6)	769 (31.0)	719 (33.0)
	wt	2,030 (62.4)	1,714 (69.0)	1,458 (67.0)
CIMP	+	1,200 (35.7)	282 (11.2)	193 (8.6)
	-	2,165 (64.3)	2,245 (88.8)	2,060 (91.4)
MSI	MSI-H	1,135 (29.5)	175 (5.6)	80 (2.8)
	MSI-L/MSS	2,710 (60.5)	2,917 (94.4)	2,737 (97.2)

* MSS: microsatellite stable; MSI: microsatellite instability; CIMP: CpG island methylation phenotype; wt: wild type; mut: mutated.

Supplementary Table 3. Association between smoking status and individual molecular subtypes of colorectal cancer, stratified by colonic location [a]

Marker	Status	N cases	Smoking									
			Never Smokers		Former Smokers				Current Smokers			
			OR	95% CI	OR	95% CI	p-value[b]	p-diff[c]	OR	95% CI	p-value[b]	p-diff[c]
Proximal colon												
BRAF	mut	827	1.00	ref	1.41	(1.18, 1.68)	1.1E-04		1.86	(1.45, 2.38)	8.5E-07	
	wt	2,848	1.00	ref	1.03	(0.94, 1.14)	0.51	4.2E-03	1.21	(1.04, 1.40)	0.01	3.7E-04
KRAS	mut	1,222	1.00	ref	1.03	(0.90, 1.19)	0.64		0.98	(0.78, 1.22)	0.85	
	wt	2,030	1.00	ref	1.15	(1.02, 1.28)	0.02	0.28	1.45	(1.23, 1.80)	8.3E-06	2.2E-03
CIMP	+	1,200	1.00	ref	1.30	(1.11, 1.51)	7.7E-04		1.85	(1.50, 2.28)	1.3E-08	
	-	2,165	1.00	ref	1.05	(0.94, 1.17)	0.42	0.04	1.09	(0.92, 1.29)	0.31	1.0E-06
MSI	MSI-H	1,135	1.00	ref	1.24	(1.06, 1.44)	6.5E-03		1.86	(1.52, 2.28)	2.1E-09	
	MSI-L/MSS	2,710	1.00	ref	1.06	(0.95, 1.17)	0.29	0.08	1.13	(0.97, 1.32)	0.11	1.5E-05
Distal colon												
BRAF	mut	157	1.00	ref	1.32	(0.91, 1.92)	0.15		1.52	(0.91, 2.53)	0.11	
	wt	2,741	1.00	ref	1.25	(1.13, 1.30)	1.3E-05	0.90	1.16	(0.99, 1.35)	0.06	0.33
KRAS	mut	769	1.00	ref	1.43	(1.20, 1.70)	5.7E-05		1.23	(0.95, 1.59)	0.12	
	wt	1,714	1.00	ref	1.18	(1.04, 1.33)	7.9E-03	0.06	1.14	(0.96, 1.37)	0.14	0.63
CIMP	+	282	1.00	ref	1.46	(1.08, 1.98)	0.02		1.42	(0.93, 2.19)	0.11	
	-	2,245	1.00	ref	1.21	(1.09, 1.35)	4.3E-04	0.37	1.13	(0.96, 1.33)	0.14	0.19
MSI	MSI-H	175	1.00	ref	1.45	(0.99, 2.13)	0.06		2.59	(1.64, 4.08)	4.2E-05	
	MSI-L/MSS	2,917	1.00	ref	1.25	(1.13, 1.38)	1.5E-05	0.41	1.13	(0.97, 1.31)	0.12	6.9E-04
Rectum												
BRAF	mut	91	1.00	ref	1.36	(0.81, 2.28)	0.24		2.18	(1.19, 3.99)	0.01	
	wt	2,596	1.00	ref	1.23	(1.10, 1.37)	2.4E-04	0.57	1.49	(1.29, 1.74)	1.4E-07	0.07
KRAS	mut	719	1.00	ref	1.16	(0.97, 1.39)	0.11		1.30	(1.00, 1.69)	0.05	
	wt	1,458	1.00	ref	1.30	(1.14, 1.48)	1.4E-04	0.29	1.50	(1.25, 1.80)	1.5E-05	0.26
CIMP	+	193	1.00	ref	1.64	(1.10, 2.43)	0.01		1.75	(1.06, 2.90)	0.03	
	-	2,060	1.00	ref	1.21	(1.07, 1.36)	1.5E-03	0.13	1.45	(1.23, 1.71)	7.2E-06	0.22
MSI	MSI-H	80	1.00	ref	0.95	(0.52, 1.76)	0.88		2.24	(1.19, 4.22)	0.01	
	MSI-L/MSS	2,737	1.00	ref	1.23	(1.11, 1.37)	1.5E-04	0.44	1.53	(1.32, 1.77)	1.1E-08	0.17

[a] MSS: microsatellite stable; MSI: microsatellite instability; CIMP: CpG island methylation phenotype; wt: wild type; mut: mutated.

[b] Two-side Wald test was used to calculate p-values. A Bonferroni corrected P value threshold of $6.3x10^{-3}$ was used for case-control analyses.

[c] Two-side Wald test was used to calculate p-values. A Bonferroni corrected P value threshold of $3.1x10^{-3}$ was used for case-only analyses.

Supplementary Table 4. Association between smoking pack-years and individual molecular subtypes of colorectal cancer, stratified by colonic location [a]

Marker	Status	N cases	Smoking (pack-years)									
			Quartile 1		Quartile 2		Quartile 3		Quartile 4			
			OR	95% CI	OR	95% CI	OR	95% CI	OR	95% CI	p-trend[b]	p-difference[c]
Proximal colon												
BRAF	mut	677	0.87	(0.63, 1.18)	1.41	(1.09, 1.83)	1.67	(1.30, 2.13)	1.91	(1.51, 2.40)	1.6E-05	
	wt	2,467	1.01	(0.87, 1.17)	0.98	(0.85, 1.14)	1.01	(0.87, 1.18)	1.23	(1.07, 1.41)	0.10	1.5E-06
KRAS	mut	1,023	1.06	(0.86, 1.31)	0.89	(0.72, 1.11)	1.00	(0.81, 1.23)	1.04	(0.85, 1.27)	0.92	
	wt	1,751	0.92	(0.76, 1.10)	1.15	(0.97, 1.36)	1.22	(1.03, 1.44)	1.49	(1.28, 1.74)	9.7E-05	5.4E-04
CIMP	+	925	1.12	(0.88, 1.42)	1.27	(1.01, 1.60)	1.48	(1.19, 1.84)	1.80	(1.47, 2.20)	2.9E-05	
	-	1,988	1.02	(0.87, 1.20)	0.98	(0.84, 1.16)	0.99	(0.84, 1.16)	1.16	(1.00, 1.36)	0.28	2.4E-07
MSI	MSI-H	946	1.06	(0.84, 1.35)	1.19	(0.95, 1.50)	1.52	(1.23, 1.88)	1.65	(1.34, 2.02)	1.1E-04	
	MSI-L/MSS	2,321	1.00	(0.86, 1.16)	1.02	(0.88, 1.19)	0.99	(0.85, 1.16)	1.18	(1.03, 1.37)	0.26	1.2E-04
Distal colon												
BRAF	mut	134	0.70	(0.34, 1.43)	1.62	(0.97, 2.71)	1.62	(0.97, 2.70)	1.75	(1.05, 2.90)	6.5E-03	
	wt	2,362	0.91	(0.77, 1.07)	1.32	(1.15, 1.53)	1.25	(1.08, 1.45)	1.47	(1.27, 1.69)	6.0E-10	0.27
KRAS	mut	663	1.07	(0.81, 1.41)	1.36	(1.05, 1.74)	1.60	(1.26, 2.03)	1.61	(1.27, 2.05)	2.2E-06	
	wt	1,473	0.82	(0.67, 1.01)	1.27	(1.07, 1.51)	1.14	(0.95, 1.36)	1.38	(1.16, 1.63)	2.1E-05	0.08
CIMP	+	192	0.79	(0.44, 1.41)	1.31	(0.81, 2.11)	1.94	(1.27, 2.96)	1.92	(1.27, 2.96)	1.4E-04	
	-	2,011	0.89	(0.75, 1.06)	1.27	(1.09, 1.49)	1.22	(1.05, 1.43)	1.41	(1.21, 1.64)	2.7E-07	0.04
MSI	MSI-H	151	1.03	(0.56, 1.93)	1.61	(0.95, 2.71)	2.21	(1.37, 3.57)	2.23	(1.37, 3.65)	1.0E-04	
	MSI-L/MSS	2,470	0.89	(0.75, 1.04)	1.29	(1.12, 1.49)	1.26	(1.09, 1.46)	1.42	(1.24, 1.64)	7.4E-09	0.02
Rectum												
BRAF	mut	75	0.57	(0.20, 1.64)	1.86	(0.98, 3.53)	1.65	(0.83, 3.29)	1.97	(1.00, 3.88)	8.9E-03	
	wt	2,173	1.07	(0.90, 1.26)	1.29	(1.11, 1.51)	1.19	(1.01, 1.40)	1.52	(1.31, 1.76)	8.7E-10	0.13
KRAS	mut	578	1.07	(0.81, 1.42)	1.28	(0.99, 1.66)	1.04	(0.79, 1.38)	1.34	(1.04, 1.73)	0.04	
	wt	1,194	1.01	(0.91, 1.25)	1.35	(1.11, 1.63)	1.24	(1.02, 1.51)	1.61	(1.34, 1.94)	1.7E-09	0.17
CIMP	+	109	0.93	(0.45, 1.93)	1.58	(0.88, 2.87)	1.72	(0.96, 3.08)	1.97	(1.12, 3.44)	6.4E-03	
	-	1,793	1.08	(0.90, 1.29)	1.29	(1.09, 1.51)	1.17	(0.98, 1.38)	1.60	(1.36, 1.87)	7.0E-10	0.27
MSI	MSI-H	70	1.30	(0.60, 2.84)	1.02	(0.45, 2.29)	0.87	(0.35, 2.15)	1.45	(0.64, 3.31)	0.48	
	MSI-L/MSS	2,252	1.10	(0.93, 1.29)	1.34	(1.15, 1.56)	1.18	(1.00, 1.38)	1.55	(1.34, 1.80)	8.6E-11	0.70

[a] MSS: microsatellite stable; MSI: microsatellite instability; CIMP: CpG island methylation phenotype; wt: wild type; mut: mutated.
[b] Two-side Wald test was used to calculate p-values. A Bonferroni corrected P value threshold of $6.3x10^{-3}$ was used for case-control analyses.
[c] Two-side Wald test was used to calculate p-values. A Bonferroni corrected P value threshold of $3.1x10^{-3}$ was used for case-only analyses.

Supplementary Table 5. Association between smoking status and individual molecular subtypes of colorectal cancer, stratified by sex [a]

Marker	Status	N cases	Smoking									
			Never Smokers		Former Smokers				Current Smokers			
			OR	95% CI	OR	95% CI	p-value[b]	p-diff[c]	OR	95% CI	p-value[b]	p-diff[c]
Male												
BRAF	mut	409	1.00	ref	1.30	(1.01, 1.67)	0.04		1.86	(1.32, 2.62)	4.3E-04	
	wt	4,687	1.00	ref	1.26	(1.15, 1.39)	7.6E-07	0.80	1.37	(1.20, 1.58)	8.2E-06	0.04
KRAS	mut	1,510	1.00	ref	1.27	(1.11, 1.45)	5.8E-04		1.15	(0.93, 1.43)	0.19	
	wt	2,821	1.00	ref	1.23	(1.10, 1.37)	1.6E-04	0.74	1.46	(1.25, 1.71)	2.8E-06	0.03
CIMP	+	755	1.00	ref	1.33	(1.09, 1.62)	4.7E-03		1.66	(1.25, 2.22)	5.6E-04	
	-	3,678	1.00	ref	1.27	(1.15, 1.40)	3.3E-06	0.69	1.38	(1.18, 1.60)	3.1E-05	0.03
MSI	MSI-H	634	1.00	ref	1.49	(1.20, 1.84)	3.3E-04		2.20	(1.67, 2.91)	2.3E-08	
	MSI-L/MSS	4,750	1.00	ref	1.26	(1.14, 1.38)	1.4E-06	0.12	1.36	(1.18, 1.56)	1.6E-05	4.8E-04
Female												
BRAF	mut	730	1.00	ref	1.53	(1.27, 1.85)	1.2E-05		1.90	(1.46, 2.47)	1.4E-06	
	wt	3,872	1.00	ref	1.06	(0.96, 1.18)	0.25	8.1E-04	1.20	(1.03, 1.38)	0.02	9.6E-04
KRAS	mut	1,327	1.00	ref	1.02	(0.88, 1.19)	0.78		1.07	(0.87, 1.32)	0.53	
	wt	2,605	1.00	ref	1.21	(1.08, 1.36)	1.3E-03	0.04	1.34	(1.14, 1.57)	3.5E-04	0.04
CIMP	+	1,013	1.00	ref	1.39	(1.17, 1.67)	2.2E-04		1.91	(1.52, 2.41)	4.5E-08	
	-	3,052	1.00	ref	1.07	(0.95, 1.19)	0.26	0.01	1.08	(0.92, 1.27)	0.33	5.5E-06
MSI	MSI-H	862	1.00	ref	1.25	(1.04, 1.50)	0.02		1.97	(1.56, 2.49)	1.6E-08	
	MSI-L/MSS	4,001	1.00	ref	1.09	(0.99, 1.21)	0.09	0.21	1.18	(1.02, 1.37)	0.02	3.9E-05

[a] MSS: microsatellite stable; MSI: microsatellite instability; CIMP: CpG island methylation phenotype; wt: wild type; mut: mutated;

[b] Two-side Wald test was used to calculate p-values. A Bonferroni corrected P value threshold of $6.3x10^{-3}$ was used for case-control analyses.

[c] Two-side Wald test was used to calculate p-values. A Bonferroni corrected P value threshold of $3.1x10^{-3}$ was used for case-only analyses.

.

Supplementary Table 6. Association between smoking pack-years and individual molecular subtypes of colorectal cancer, stratified by sex [a]

			Smoking (pack-years)									
Marker	Status	N cases	Quartile 1		Quartile 2		Quartile 3		Quartile 4			
			OR	95% CI	OR	95% CI	OR	95% CI	OR	95% CI	p-trend[b]	p-difference[c]
Male												
BRAF	mut	326	0.88	(0.58, 1.34)	1.47	(1.04, 2.09)	1.59	(1.13, 2.24)	1.71	(1.23, 2.38)	8.4E-05	
	wt	3,895	1.07	(0.93, 1.23)	1.27	(1.12, 1.45)	1.29	(1.13, 1.48)	1.49	(1.31, 1.48)	3.5E-11	0.15
KRAS	mut	1,218	1.18	(0.96, 1.44)	1.20	(0.99, 1.44)	1.31	(1.08, 1.59)	1.32	(1.09, 1.60)	7.7E-04	
	wt	2,346	0.95	(0.81, 1.13)	1.31	(1.12, 1.52)	1.28	(1.10, 1.49)	1.52	(1.32, 1.76)	4.4E-10	0.13
CIMP	+	503	0.99	(0.72, 1.36)	1.27	(0.95, 1.71)	1.61	(1.22, 2.12)	1.69	(1.29, 2.21)	3.9E-06	
	-	3,216	1.09	(0.94, 1.26)	1.33	(1.16, 1.53)	1.30	(1.13, 1.50)	1.49	(1.30, 1.71)	2.6E-10	0.10
MSI	MSI-H	515	1.39	(1.02, 1.90)	1.41	(1.04, 1.91)	1.94	(1.47, 2.58)	2.01	(1.51, 2.67)	2.9E-08	
	MSI-L/MSS	3,854	1.06	(0.92, 1.22)	1.29	(1.13, 1.47)	1.26	(1.13, 1.47)	1.45	(1.28, 1.65)	5.0E-10	6.1E-03
Female												
BRAF	mut	595	0.80	(0.56, 1.15)	1.61	(1.22, 2.14)	1.75	(1.33, 2.31)	2.11	(1.63, 2.72)	3.4E-11	
	wt	3,338	0.91	(0.77, 1.08)	1.15	(0.98, 1.34)	1.01	(0.86, 1.19)	1.23	(1.05, 1.43)	0.02	1.9E-07
KRAS	mut	1,113	0.89	(0.70, 1.13)	1.03	(0.82, 1.29)	0.98	(0.78, 1.24)	1.14	(0.92, 1.42)	0.36	
	wt	2,201	0.91	(0.75, 1.10)	1.27	(1.06, 1.51)	1.18	(0.98, 1.41)	1.45	(1.22, 1.71)	3.6E-06	0.01
CIMP	+	744	1.10	(0.81, 1.47)	1.36	(1.03, 1.78)	1.56	(1.20, 2.02)	2.04	(1.61, 2.58)	3.8E-10	
	-	2,750	0.91	(0.76, 1.09)	1.08	(0.91, 1.28)	0.99	(0.83, 1.18)	1.22	(1.04, 1.44)	0.05	2.9E-06
MSI	MSI-H	724	0.99	(0.73, 1.35)	1.31	(1.00, 1.73)	1.46	(1.12, 1.90)	1.70	(1.33, 2.18)	2.1E-06	
	MSI-L/MSS	3,401	0.90	(0.76, 1.06)	1.18	(1.01, 1.39)	1.03	(0.88, 1.22)	1.27	(1.08, 1.48)	3.6E-03	3.1E-03

[a] MSS: microsatellite stable; MSI: microsatellite instability; CIMP: CpG island methylation phenotype; wt: wild type; mut: mutated;

[b] Two-side Wald test was used to calculate p-values. A Bonferroni corrected P value threshold of 6.3×10^{-3} was used for case-control analyses.

[c] Two-side Wald test was used to calculate p-values. A Bonferroni corrected P value threshold of 3.1×10^{-3} was used for case-only analyses.

Supplementary Table 7. Association between smoking status and individual molecular subtypes of colorectal cancer, stratified by study design [a]

Marker	Status	N cases	Never Smokers		Former Smokers				Current Smokers			
			OR	95% CI	OR	95% CI	p-value[b]	p-diff[c]	OR	95% CI	p-value[b]	p-diff[c]
Cohort												
BRAF	mut	373	1.00	ref	1.23	(0.97, 1.56)	0.08		1.76	(1.21, 2.56)	2.9E-03	
	wt	2,099	1.00	ref	1.19	(1.06, 1.35)	4.97E-03	0.76	1.43	(1.15, 1.77)	1.1E-03	0.30
KRAS	mut	824	1.00	ref	1.21	(1.02, 1.43)	0.03		1.22	(0.90, 1.64)	0.20	
	wt	1,526	1.00	ref	1.17	(1.02, 1.34)	0.03	0.80	1.52	(1.21, 1.91)	3.7E-04	0.12
CIMP	+	427	1.00	ref	1.29	(1.03, 1.62)	0.03		2.36	(1.67, 3.34)	1.4E-06	
	-	2,094	1.00	ref	1.17	(1.04, 1.33)	0.01	0.42	1.31	(1.06, 1.68)	0.02	1.3E-03
MSI	MSI-H	400	1.00	ref	1.33	(1.06, 1.68)	0.02		2.19	(1.54, 3.14)	1.6E-05	
	MSI-L/MSS	2,066	1.00	ref	1.17	(1.03, 1.32)	0.02	0.26	1.38	(1.11, 1.71)	3.7E-03	0.14
Case-control												
BRAF	mut	635	1.00	ref	1.59	(1.30, 1.93)	4.1E-06		2.05	(1.59, 2.64)	2.4E-08	
	wt	5,596	1.00	ref	1.16	(1.07, 1.26)	4.8E-04	4.4E-03	1.25	(1.11, 1.40)	1.6E-04	2.7E-05
KRAS	mut	1,660	1.00	ref	1.13	(1.00, 1.28)	0.05		1.08	(0.91, 1.29)	0.38	
	wt	3,366	1.00	ref	1.23	(1.12, 1.36)	1.6E-05	0.21	1.38	(1.21, 1.57)	1.0E-06	9.0E-03
CIMP	+	955	1.00	ref	1.42	(1.21, 1.66)	2.0E-05		1.68	(1.36, 2.07)	1.6E-06	
	-	4,138	1.00	ref	1.16	(1.06, 1.27)	1.4E-03	0.03	1.21	(1.07, 1.38)	2.9E-03	2.6E-04
MSI	MSI-H	934	1.00	ref	1.24	(1.05, 1.47)	0.01		1.96	(1.59, 2.40)	1.3E-10	
	MSI-L/MSS	5,762	1.00	ref	1.18	(1.09, 1.28)	5.1E-05	0.58	1.25	(1.11, 1.40)	1.2E-04	9.5E-06

Smoking: Never Smokers, Former Smokers, Current Smokers.

[a] MSS: microsatellite stable; MSI: microsatellite instability; CIMP: CpG island methylation phenotype; wt: wild type; mut: mutated;

[b] Two-side Wald test was used to calculate p-values. A Bonferroni corrected P value threshold of $6.3x10^{-3}$ was used for case-control analyses.

[c] Two-side Wald test was used to calculate p-values. A Bonferroni corrected P value threshold of $3.1x10^{-3}$ was used for case-only analyses.

Supplementary Table 8. Association between smoking pack-years and individual molecular subtypes of colorectal cancer, stratified by study design [a]

Marker	Status	N cases	Quartile 1 OR	Quartile 1 95% CI	Quartile 2 OR	Quartile 2 95% CI	Quartile 3 OR	Quartile 3 95% CI	Quartile 4 OR	Quartile 4 95% CI	p-trend[b]	p-difference[c]
Cohort												
BRAF	mut	366	0.78	(0.51, 1.19)	1.34	(0.95, 1.89)	1.14	(0.80, 1.62)	2.04	(1.52, 2.74)	1.3E-05	
	wt	2,022	1.05	(0.86, 1.27)	1.20	(1.00, 1.44)	1.15	(0.96, 1.38)	1.46	(1.23, 1.74)	3.0E-05	0.04
KRAS	mut	792	1.11	(0.86, 1.44)	1.11	(0.86, 1.43)	1.09	(0.84, 1.40)	1.49	(1.18, 1.88)	3.7E-03	
	wt	1,475	0.92	(0.74, 1.14)	1.26	(1.03, 1.53)	1.18	(0.96, 1.44)	1.48	(1.23, 1.79)	2.1E-05	0.51
CIMP	+	413	1.07	(0.74, 1.54)	1.13	(0.80, 1.60)	1.27	(0.91, 1.77)	2.18	(1.64, 2.89)	8.8E-07	
	-	2,026	1.01	(0.84, 1.22)	1.19	(1.00, 1.43)	1.14	(0.95, 1.37)	1.39	(1.17, 1.65)	1.8E-04	5.6E-03
MSI	MSI-H	395	1.15	(0.80, 1.66)	1.29	(0.91, 1.82)	1.36	(0.97, 1.90)	2.16	(1.62, 2.89)	1.1E-06	
	MSI-L/MSS	1,989	1.00	(0.83, 1.22)	1.18	(0.98, 1.42)	1.12	(0.93, 1.35)	1.43	(1.20, 1.70)	1.1E-04	8.1E-03
Case-control												
BRAF	mut	555	0.90	(0.63, 1.29)	1.74	(1.31, 2.31)	2.23	(1.70, 2.91)	1.82	(1.38, 2.39)	2.7E-10	
	wt	5,211	0.96	(0.85, 1.09)	1.21	(1.07, 1.36)	1.17	(1.04, 1.33)	1.33	(1.18, 1.50)	1.5E-07	2.0E-05
KRAS	mut	1,539	1.01	(0.84, 1.22)	1.12	(0.94, 1.34)	1.20	(1.01, 1.44)	1.10	(0.92, 1.32)	0.06	
	wt	3,072	0.93	(0.80, 1.09)	1.29	(1.12, 1.48)	1.26	(1.09, 1.45)	1.48	(1.29, 1.69)	1.4E-10	6.6E-03
CIMP	+	834	1.05	(0.80, 1.38)	1.44	(1.13, 1.84)	1.83	(1.46, 2.31)	1.72	(1.37, 2.15)	6.8E-10	
	-	3,940	0.99	(0.86, 1.14)	1.21	(1.06, 1.38)	1.16	(1.02, 1.33)	1.34	(1.18, 1.52)	1.5E-06	4.4E-04
MSI	MSI-H	844	1.09	(0.84, 1.42)	1.29	(1.01, 1.64)	1.72	(1.37, 2.16)	1.47	(1.15, 1.86)	2.7E-06	
	MSI-L/MSS	5,266	0.97	(0.86, 1.11)	1.26	(1.12, 1.42)	1.18	(1.04, 1.33)	1.34	(1.19, 1.51)	3.8E-08	0.04

Smoking (pack-years)

[a] MSS: microsatellite stable; MSI: microsatellite instability; CIMP: CpG island methylation phenotype; wt: wild type; mut: mutated;

[b] Two-side Wald test was used to calculate p-values. A Bonferroni corrected P value threshold of $6.3x10^{-3}$ was used for case-control analyses.

[c] Two-side Wald test was used to calculate p-values. A Bonferroni corrected P value threshold of $3.1x10^{-3}$ was used for case-only analyses.

Supplementary Figure 1. Sample size of smoking status by CRC subtypes defined by combined marker status

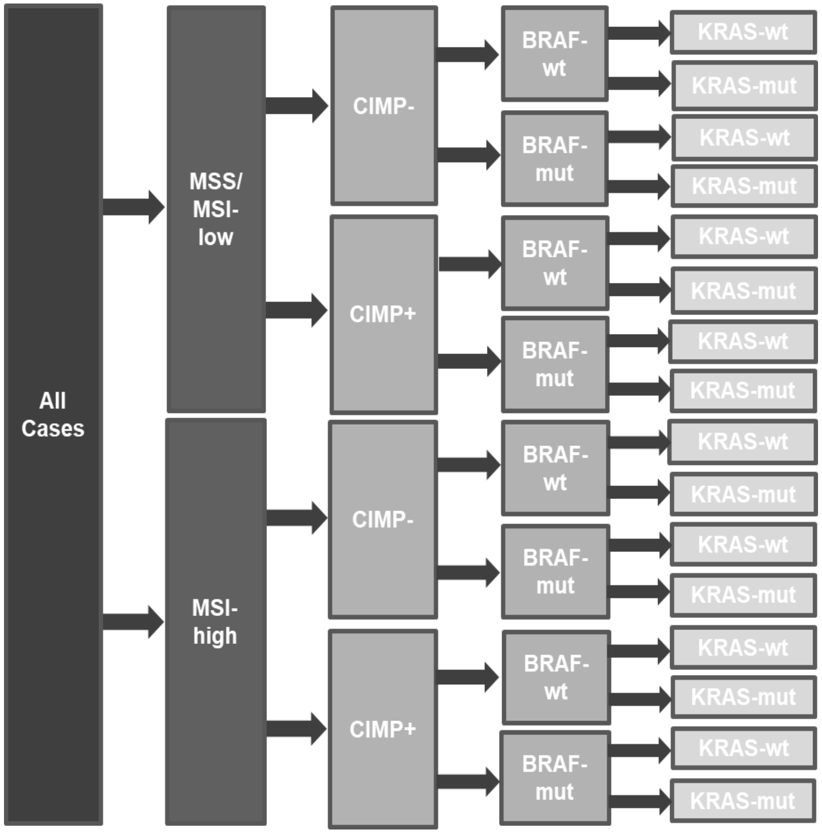

Groups	N cases	Smoking			Smoking (pack-years)				
		Never	Former	Current	Non-smoker	Q1	Q2	Q3	Q4
**Type 4**	**2,789**	1,176	1,261	352	1,161	299	400	352	439
**Type 3**	**1,680**	742	752	186	736	200	205	228	241
**Type 6**	**159**	76	60	23	76	8	18	19	32
Type 7	23	4	16	3	2	2	7	4	5
**Type 8**	**208**	85	83	40	76	20	18	26	28
**Type 9**	**236**	101	106	29	96	30	31	26	32
**Type 2**	**157**	69	57	31	66	4	21	23	22
Type 10	4	3	1	0	2	0	0	1	0
**Type 5**	**188**	81	60	47	81	21	23	34	22
**Type 11**	**90**	47	34	9	47	10	8	13	11
Type 12	36	15	18	3	15	3	6	7	5
Type 13	3	1	2	0	1	1	1	0	0
**Type 14**	**138**	47	58	33	46	13	14	18	36
Type 15	34	15	15	4	14	2	2	7	8
**Type 1**	**389**	154	187	48	150	36	47	57	76
Type 16	10	5	3	2	5	0	0	3	2

MSS: microsatellite stable; MSI: microsatellite instability; CIMP: CpG island methylation phenotype; wt: wild type; mut: mutated;
Bold types are the CRC subtypes with at least 50 cases and were included in the combined marker analysis.

Supplementary Figure 2. Association between former and current smokers and risk of CRC subtypes defined by combined marker status, stratified by colonic location

A) Proximal colon: Former smokers vs. never smokers

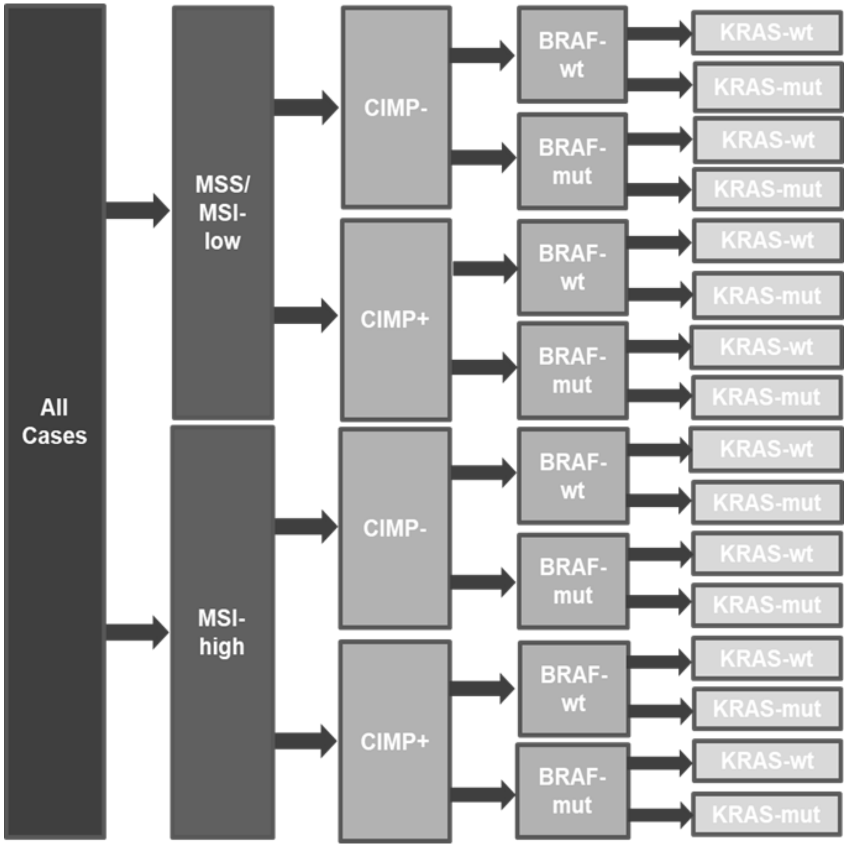

Subtypes	N Cases	OR	95% CI	p-value	p-difference
Type 4	312	1.23	(1.03,1.46)	2.18E-02	reference
Type 3	294	1.05	(0.88,1.25)	5.91E-01	1.73E-01
Type 6	25	0.65	(0.39,1.08)	9.53E-02	5.12E-02
Type 7	7				
Type 8	27	0.90	(0.54,1.48)	6.68E-01	1.96E-01
Type 9	60	1.09	(0.75,1.59)	6.38E-01	6.87E-01
Type 2	40	1.20	(0.77,1.88)	4.23E-01	7.91E-01
Type 10	1				
Type 5	39	0.84	(0.54,1.31)	4.39E-01	1.01E-01
Type 11	20	0.73	(0.40,1.31)	2.88E-01	6.71E-02
Type 12	16				
Type 13	2				
Type 14	50	1.65	(1.07,2.54)	2.29E-02	1.99E-01
Type 15	11				
Type 1	163	1.59	(1.24,2.04)	2.23E-04	6.46E-02
Type 16	3				

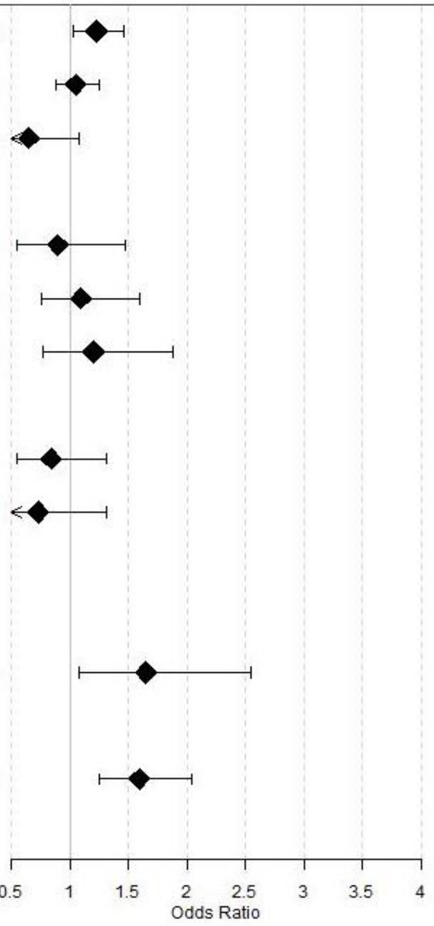

B) Proximal colon: Current smokers vs. never smokers

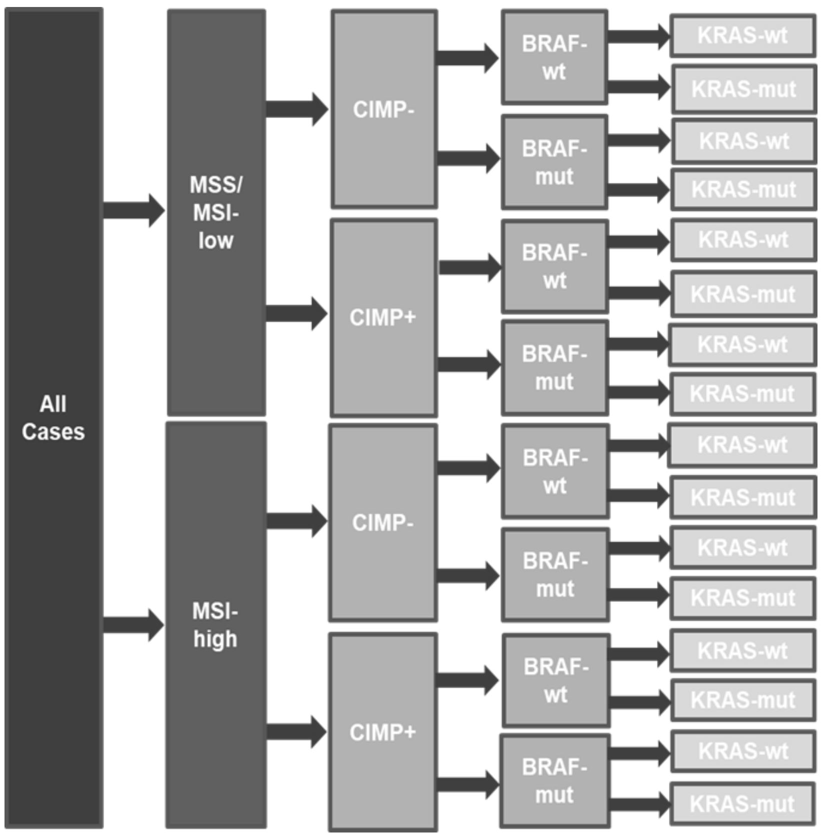

Subtypes	N Cases	OR	95% CI	p-value	p-difference
Type 4	68	1.03	(0.78,1.37)	8.13E-01	reference
Type 3	66	0.90	(0.67,1.20)	4.68E-01	4.58E-01
Type 6	8	0.84	(0.39,1.79)	6.44E-01	7.32E-01
Type 7	2				
Type 8	13	1.25	(0.66,2.38)	4.97E-01	2.44E-01
Type 9	16	1.09	(0.62,1.93)	7.60E-01	4.76E-01
Type 2	19	1.87	(1.06,3.30)	3.07E-02	2.82E-02
Type 10	0				
Type 5	30	2.02	(1.25,3.25)	3.91E-03	1.68E-02
Type 11	4	0.48	(0.16,1.39)	1.76E-01	7.54E-02
Type 12	1				
Type 13	0				
Type 14	30	4.00	(2.45,6.54)	3.30E-08	6.96E-07
Type 15	4				
Type 1	43	1.82	(1.26,2.63)	1.52E-03	3.05E-03
Type 16	2				

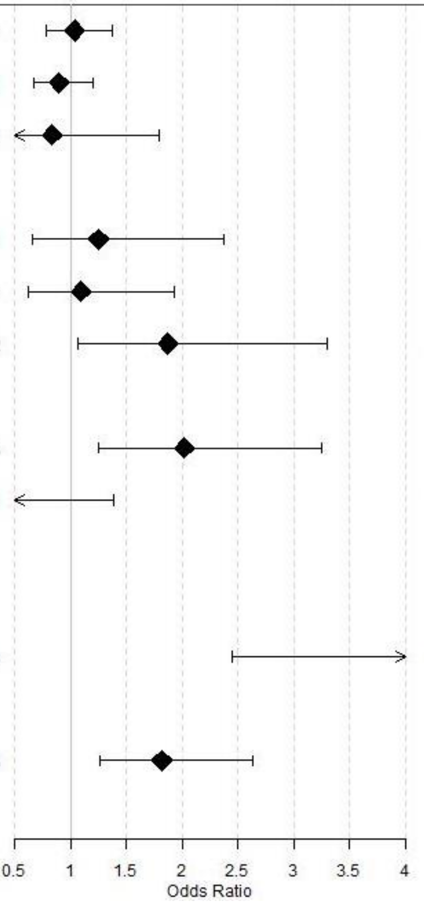

C) Distal colon: Former smokers vs. never smokers

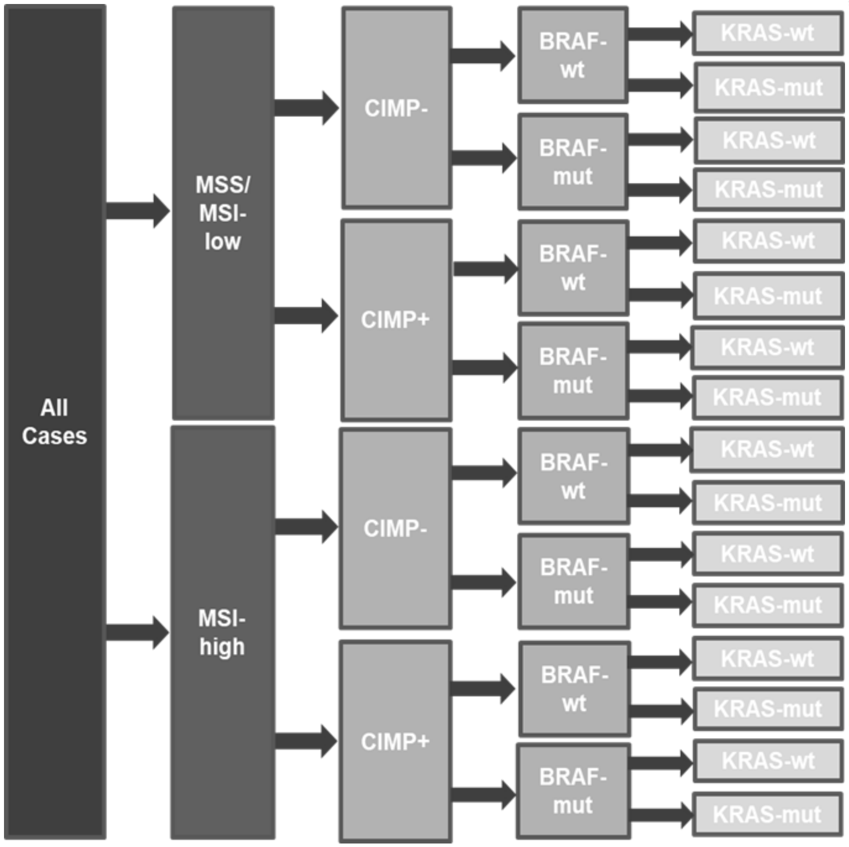

Subtypes	N Cases	OR	95% CI	p-value	p-difference
Type 4	486	1.18	(1.03,1.36)	2.00E-02	reference
Type 3	248	1.43	(1.17,1.75)	4.88E-04	1.01E-01
Type 6	17	1.32	(0.64,2.73)	4.57E-01	6.49E-01
Type 7	3				
Type 8	32	1.71	(0.98,2.98)	5.80E-02	4.57E-01
Type 9	24	1.61	(0.87,2.98)	1.33E-01	4.07E-01
Type 2	11	0.82	(0.38,1.74)	5.99E-01	3.83E-01
Type 10	0				
Type 5	9				
Type 11	6	0.96	(0.39,2.38)	9.29E-01	9.93E-01
Type 12	1				
Type 13	0				
Type 14	7	0.82	(0.05,13.76)	8.92E-01	4.46E-01
Type 15	3				
Type 1	16	1.39	(0.25,7.65)	7.05E-01	1.56E-01
Type 16	0				

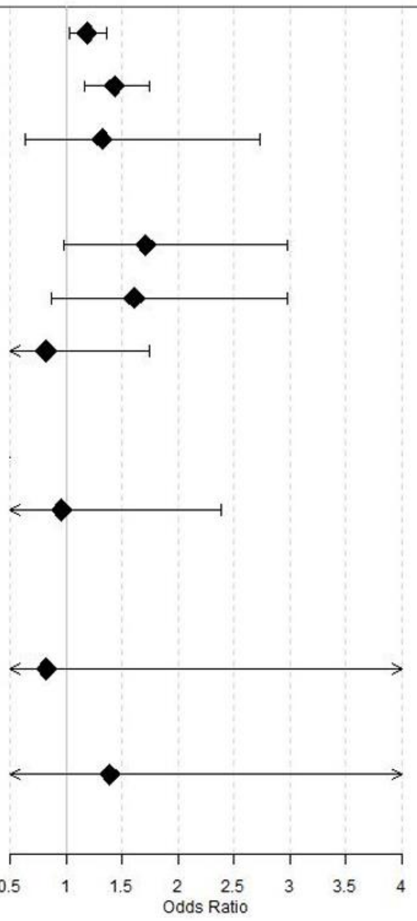

D) Distal colon: Current smokers vs. never smokers

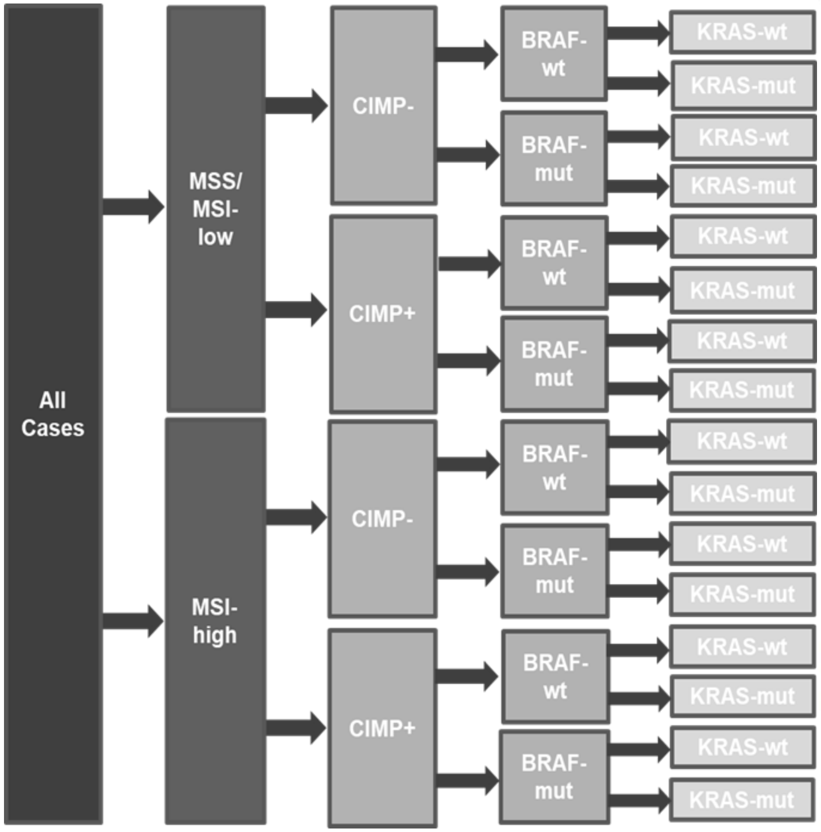

Subtypes	N Cases	OR	95% CI	p-value	p-difference
Type 4	120	0.97	(0.78,1.21)	8.05E-01	reference
Type 3	55	1.06	(0.77,1.46)	7.11E-01	6.00E-01
Type 6	8	2.16	(0.89,5.22)	8.88E-02	1.51E-01
Type 7	0				
Type 8	15	2.22	(1.13,4.38)	2.14E-02	2.57E-02
Type 9	6	1.26	(0.50,3.16)	6.28E-01	4.33E-01
Type 2	4	0.74	(0.25,2.22)	5.95E-01	6.80E-01
Type 10	0				
Type 5	6				
Type 11	4	2.04	(0.73,5.67)	1.73E-01	6.73E-02
Type 12	1				
Type 13	0				
Type 14	3	3.74	(0.26,53.05)	3.30E-01	8.94E-02
Type 15	0				
Type 1	2				
Type 16	0				

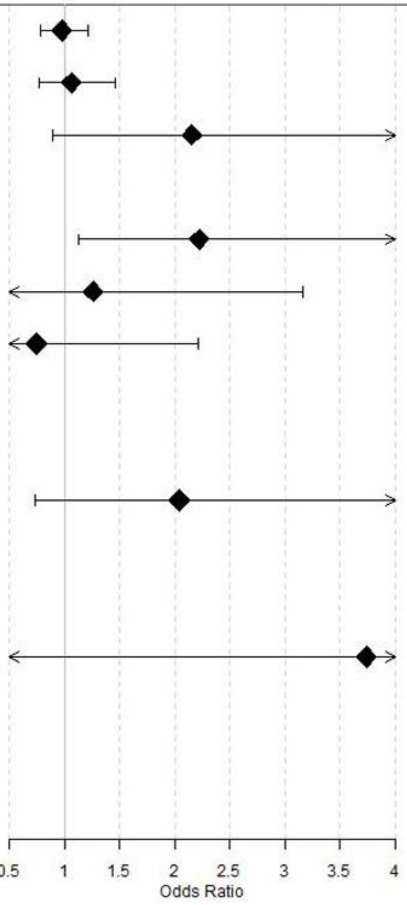

E) Rectum: Former smokers vs. never smokers

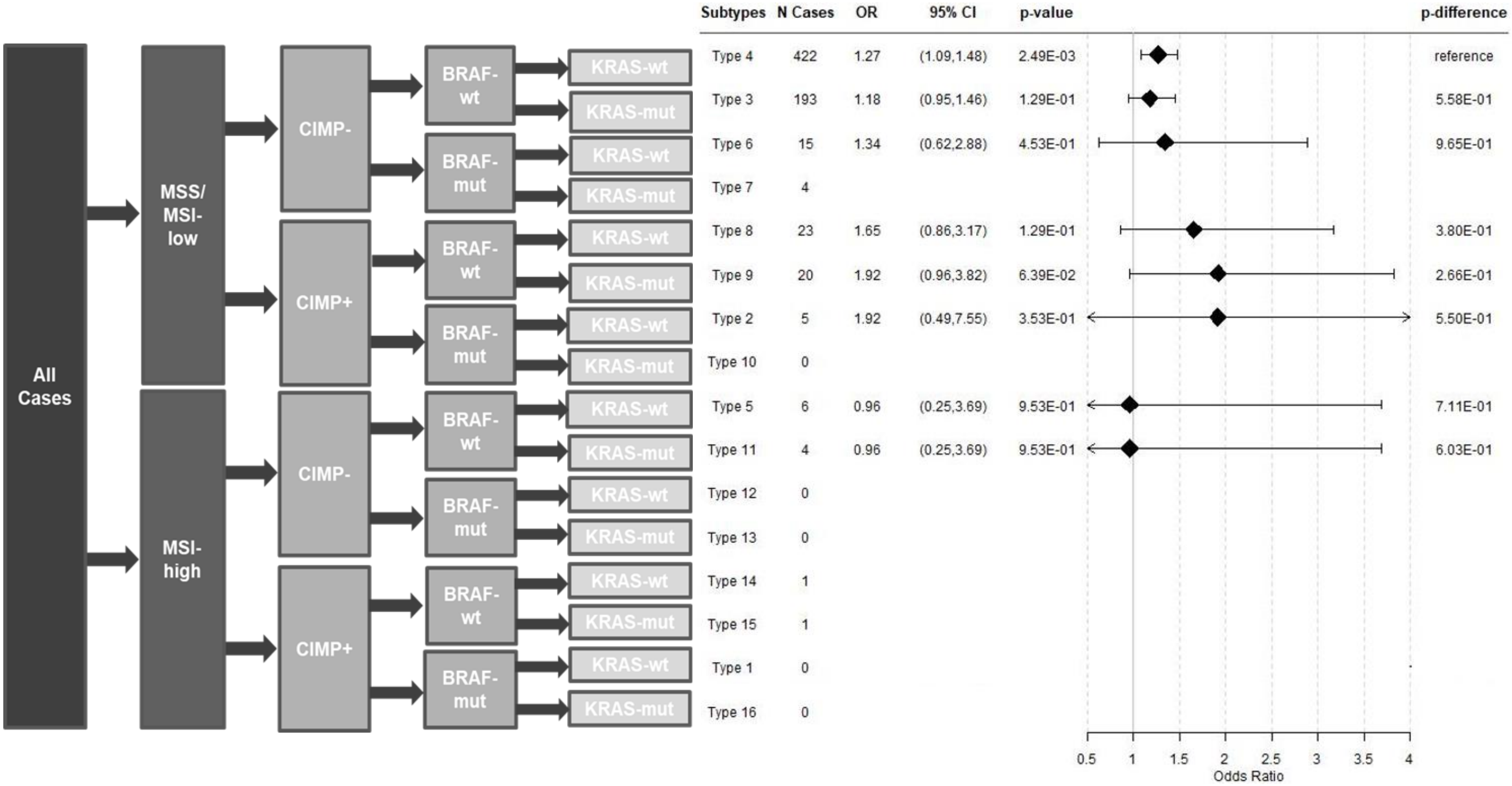

Subtypes	N Cases	OR	95% CI	p-value	p-difference
Type 4	422	1.27	(1.09,1.48)	2.49E-03	reference
Type 3	193	1.18	(0.95,1.46)	1.29E-01	5.58E-01
Type 6	15	1.34	(0.62,2.88)	4.53E-01	9.65E-01
Type 7	4				
Type 8	23	1.65	(0.86,3.17)	1.29E-01	3.80E-01
Type 9	20	1.92	(0.96,3.82)	6.39E-02	2.66E-01
Type 2	5	1.92	(0.49,7.55)	3.53E-01	5.50E-01
Type 10	0				
Type 5	6	0.96	(0.25,3.69)	9.53E-01	7.11E-01
Type 11	4	0.96	(0.25,3.69)	9.53E-01	6.03E-01
Type 12	0				
Type 13	0				
Type 14	1				
Type 15	1				
Type 1	0				
Type 16	0				

F) Rectum: Current smokers vs. never smokers

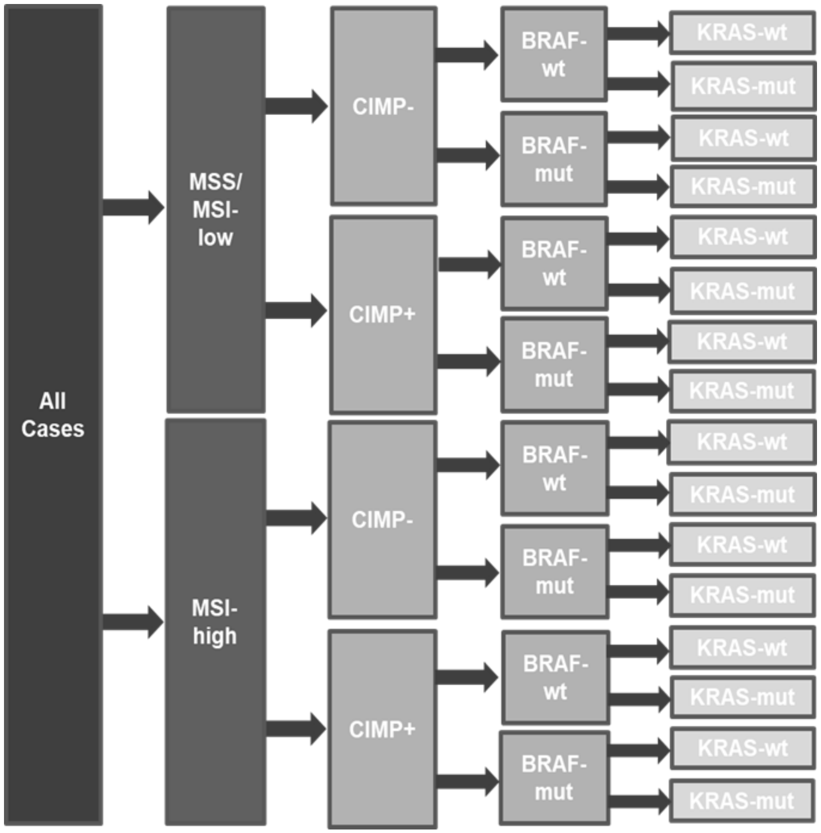

Subtypes	N Cases	OR	95% CI	p-value	p-difference
Type 4	147	1.52	(1.23,1.88)	1.23E-04	reference
Type 3	62	1.29	(0.95,1.75)	1.03E-01	3.80E-01
Type 6	7	2.09	(0.83,5.23)	1.15E-01	5.46E-01
Type 7	1				
Type 8	11	1.76	(0.79,3.91)	1.65E-01	2.73E-01
Type 9	7	1.80	(0.71,4.59)	2.17E-01	4.70E-01
Type 2	4	3.86	(0.92,16.28)	6.56E-02	1.05E-01
Type 10	0				
Type 5	7	0.68	(0.07,6.43)	7.38E-01	2.33E-01
Type 11	1	0.68	(0.07,6.43)	7.38E-01	4.74E-01
Type 12	0				
Type 13	0				
Type 14	0				
Type 15	0				
Type 1	0				
Type 16	0				

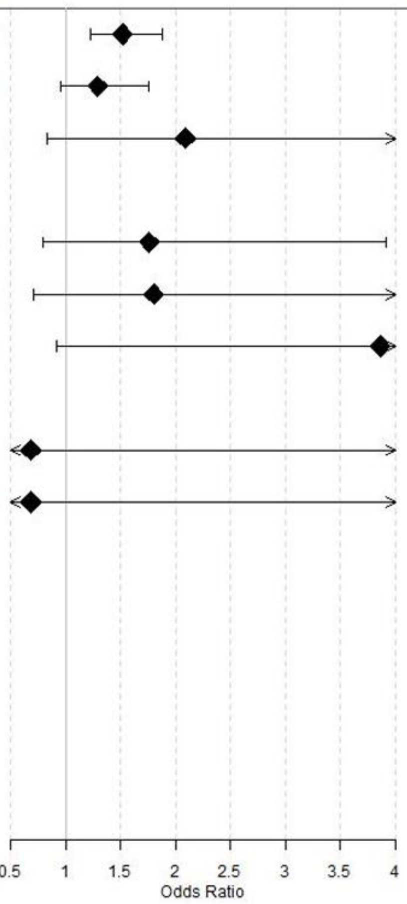

MSS: microsatellite stable; MSI: microsatellite instability; CIMP: CpG island methylation phenotype; wt: wild type; mut: mutated;OR: odds ratio; 95% CI: 95% confidence interval; Two-sided Wald test was used to calculate the P values from the case-control analysis (N controls = 11,231) and case-only analysis (Pdifference). A Bonferroni corrected p-value threshold of $5.0x10^{-3}$ was used for both case-control and case-only analyses.

Supplementary Figure 3. Associations between smoking pack-years and risk of CRC subtypes defined by combined marker status, stratified by colonic location

A) Proximal colon

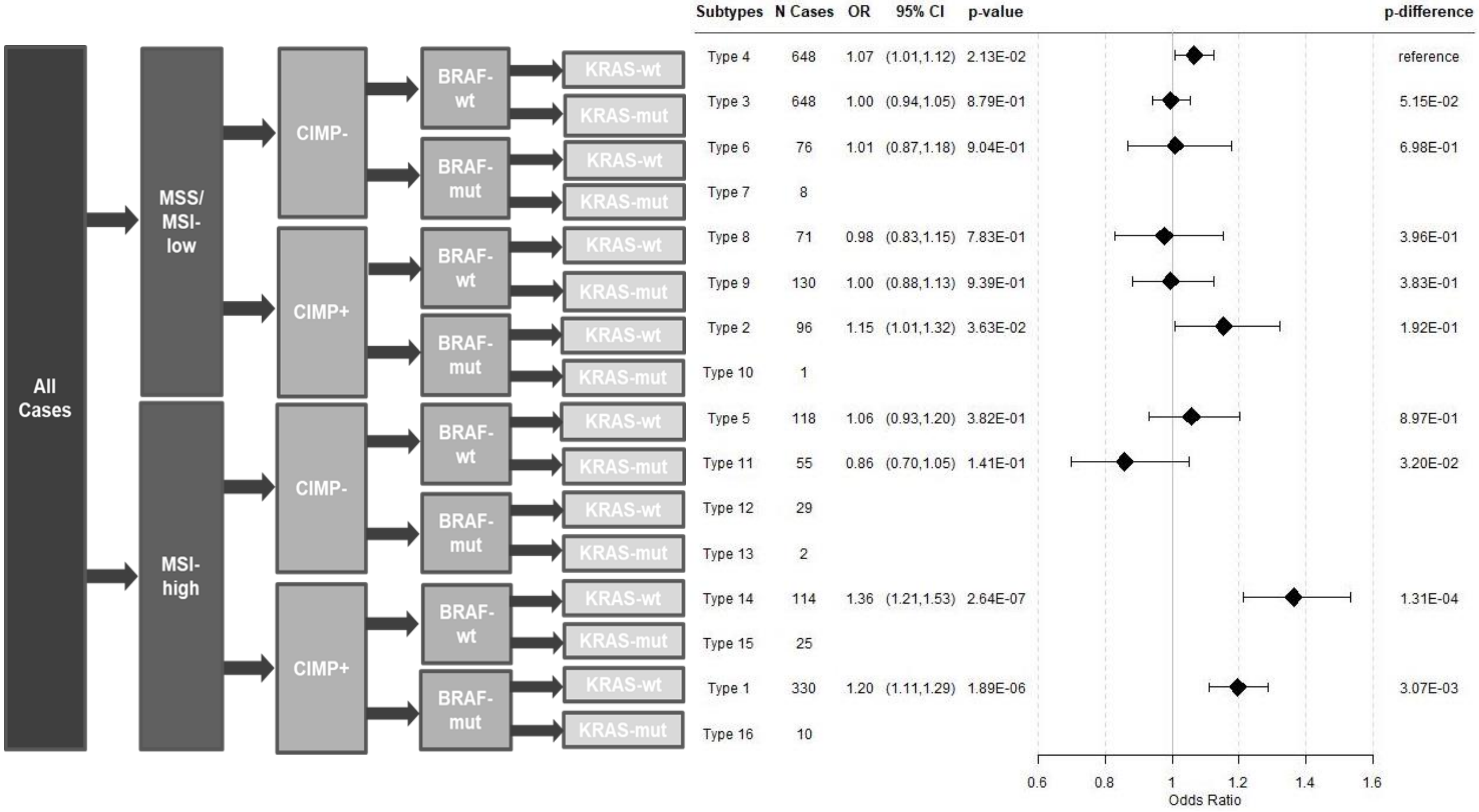

Subtypes	N Cases	OR	95% CI	p-value	p-difference
Type 4	648	1.07	(1.01,1.12)	2.13E-02	reference
Type 3	648	1.00	(0.94,1.05)	8.79E-01	5.15E-02
Type 6	76	1.01	(0.87,1.18)	9.04E-01	6.98E-01
Type 7	8				
Type 8	71	0.98	(0.83,1.15)	7.83E-01	3.96E-01
Type 9	130	1.00	(0.88,1.13)	9.39E-01	3.83E-01
Type 2	96	1.15	(1.01,1.32)	3.63E-02	1.92E-01
Type 10	1				
Type 5	118	1.06	(0.93,1.20)	3.82E-01	8.97E-01
Type 11	55	0.86	(0.70,1.05)	1.41E-01	3.20E-02
Type 12	29				
Type 13	2				
Type 14	114	1.36	(1.21,1.53)	2.64E-07	1.31E-04
Type 15	25				
Type 1	330	1.20	(1.11,1.29)	1.89E-06	3.07E-03
Type 16	10				

B) Distal colon

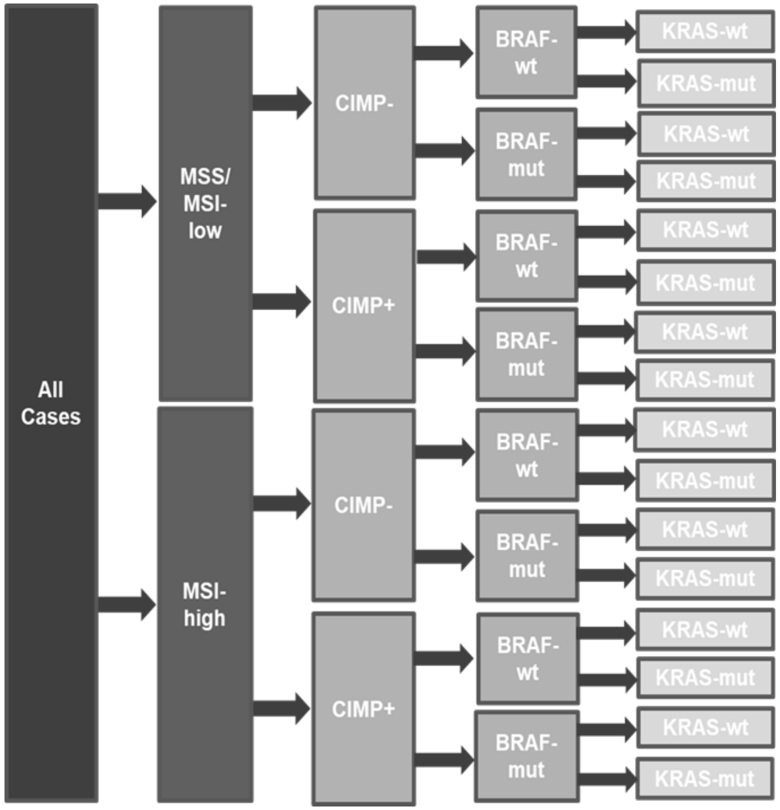

Subtypes	N Cases	OR	95% CI	p-value	p-difference
Type 4	1042	1.07	(1.02,1.12)	3.74E-03	reference
Type 3	487	1.12	(1.05,1.19)	2.85E-04	1.96E-01
Type 6	39	1.22	(0.99,1.50)	6.19E-02	2.53E-01
Type 7	3				
Type 8	58	1.23	(1.03,1.45)	1.96E-02	1.29E-01
Type 9	44	1.28	(1.05,1.55)	1.25E-02	9.02E-02
Type 2	28	1.00	(0.77,1.31)	9.88E-01	6.04E-01
Type 10	2				
Type 5	26	1.14	(0.88,1.47)	3.16E-01	6.03E-01
Type 11	15	1.44	(1.02,2.03)	3.62E-02	1.19E-01
Type 12	4				
Type 13	0				
Type 14	13	1.32	(0.93,1.86)	1.22E-01	2.87E-01
Type 15	6				
Type 1	26	1.41	(1.10,1.81)	6.62E-03	3.80E-02
Type 16	0				

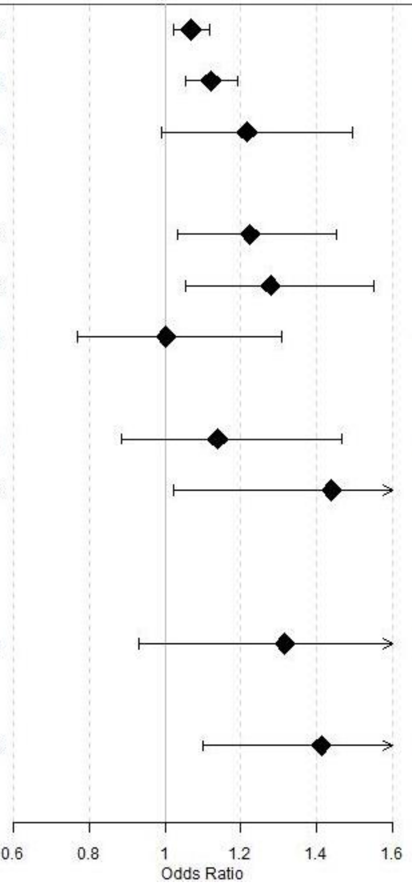

C) Rectum

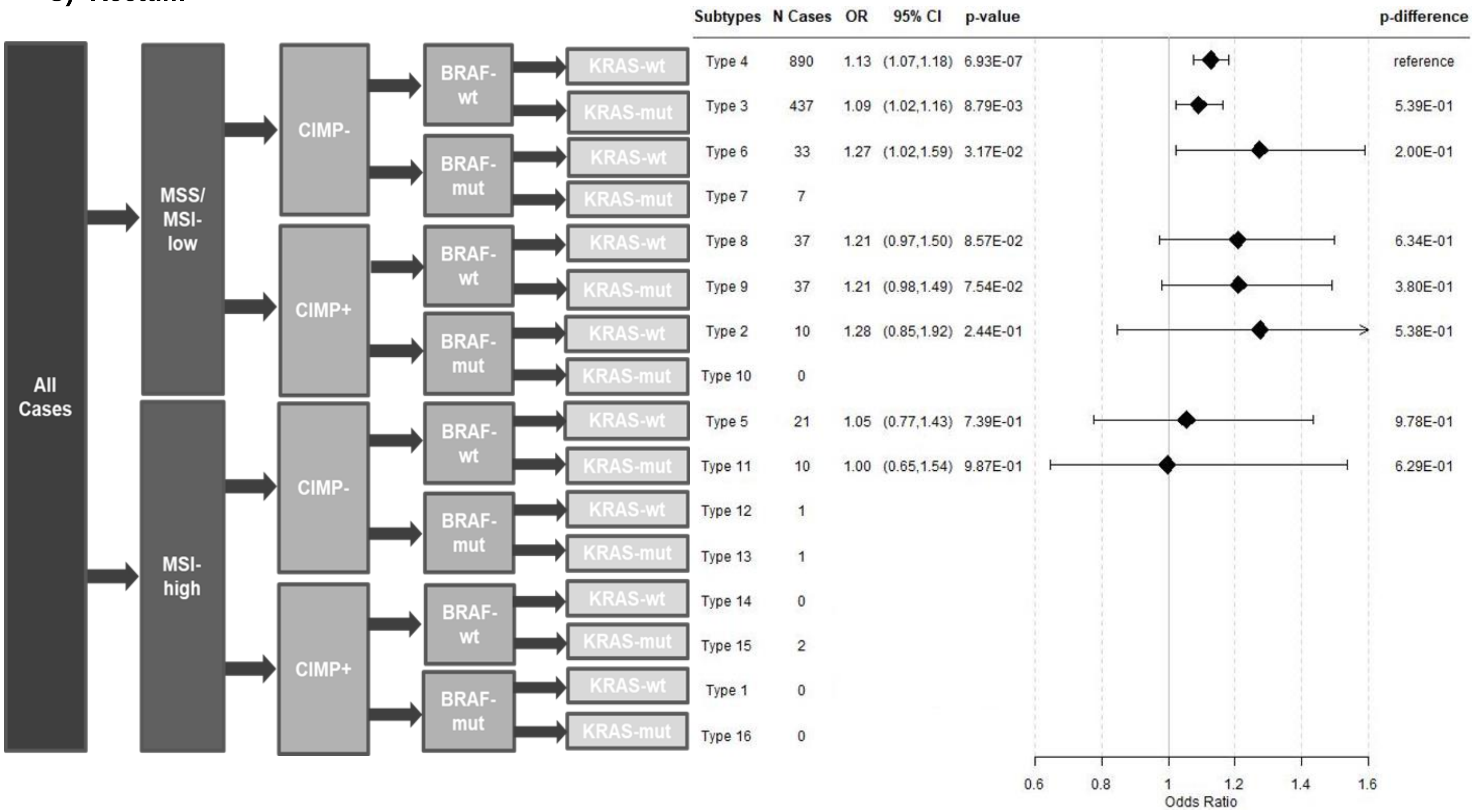

MSS: microsatellite stable; MSI: microsatellite instability; CIMP: CpG island methylation phenotype; wt: wild type; mut: mutated;OR: odds ratio; 95% CI: 95% confidence interval; Two-sided Wald test was used to calculate the P values from the case-control analysis (N controls = 10,199) and case-only analysis (Pdifference). A Bonferroni corrected p-value threshold of 5.0×10^{-3} was used for both case-control and case-only analyses.

Supplementary Figure 4. Association between former and current smokers and risk of CRC subtypes defined by combined marker status, stratified by sex

A) Male: Former smokers vs. never smokers

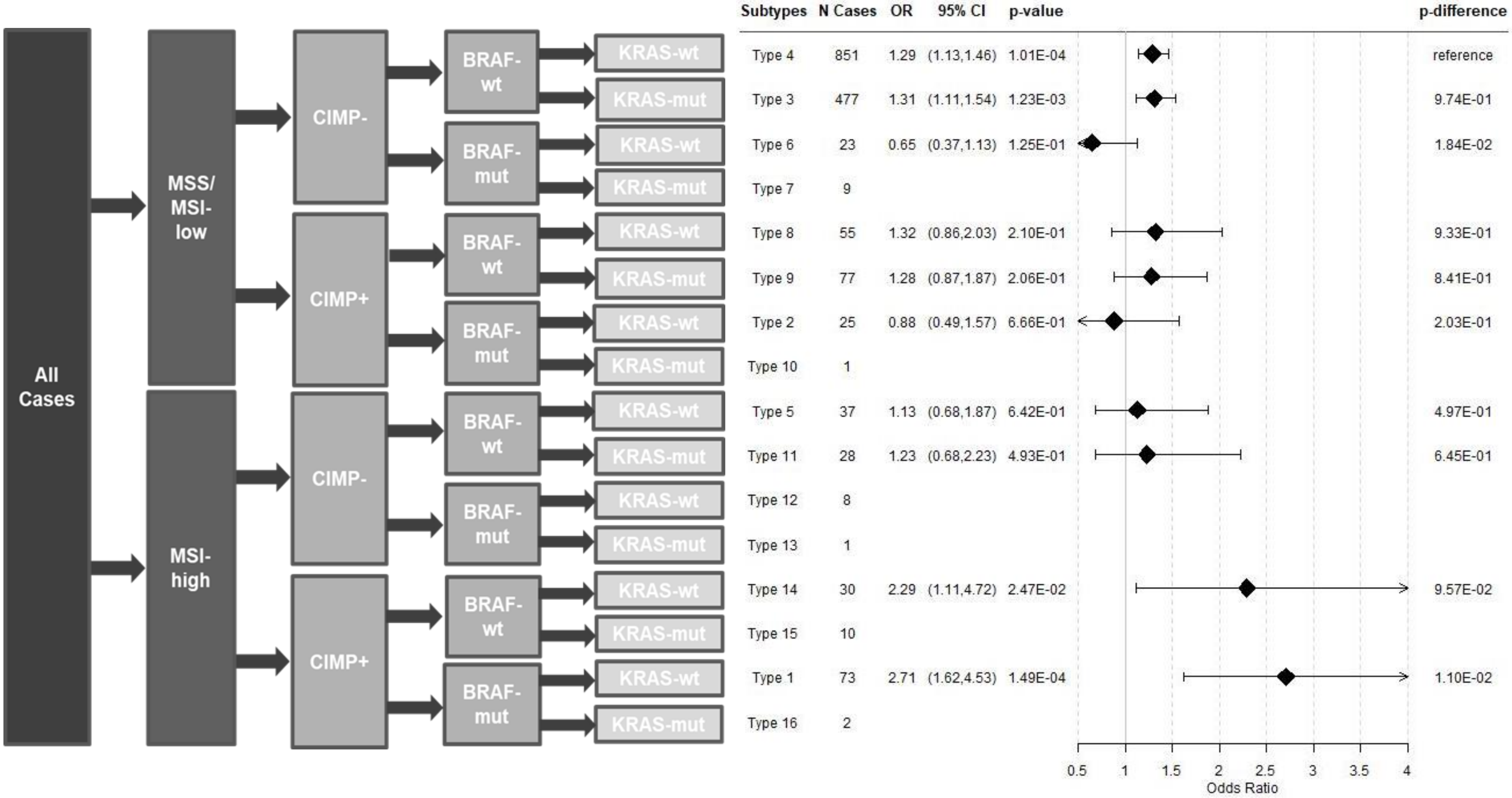

B) Male: Current smokers vs. never smokers

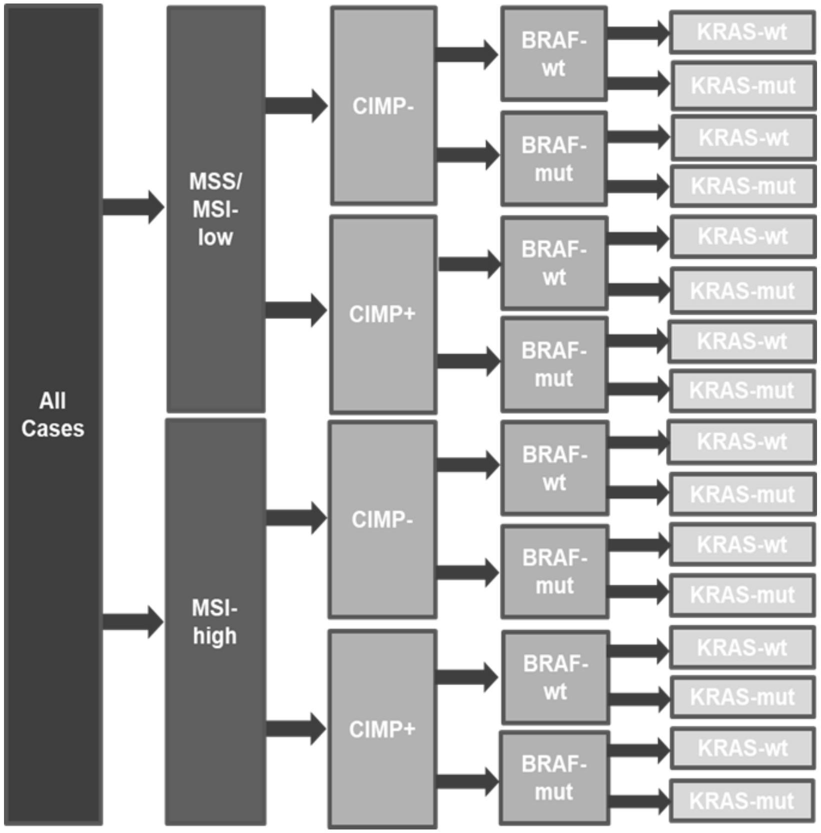

Subtypes	N Cases	OR	95% CI	p-value	p-difference
Type 4	207	1.32	(1.09,1.61)	4.33E-03	reference
Type 3	89	1.08	(0.82,1.41)	5.84E-01	1.91E-01
Type 6	12	1.38	(0.69,2.76)	3.61E-01	9.74E-01
Type 7	1				
Type 8	24	2.13	(1.24,3.67)	6.13E-03	6.37E-03
Type 9	15	1.24	(0.68,2.27)	4.74E-01	5.46E-01
Type 2	8	1.17	(0.52,2.65)	7.03E-01	8.30E-01
Type 10					
Type 5	27	2.41	(1.37,4.24)	2.21E-03	3.10E-02
Type 11	9	1.42	(0.63,3.17)	3.97E-01	6.91E-01
Type 12	2				
Type 13					
Type 14	9	3.88	(1.55,9.72)	3.77E-03	4.28E-03
Type 15	2				
Type 1	12	2.65	(1.24,5.65)	1.19E-02	2.16E-02
Type 16					

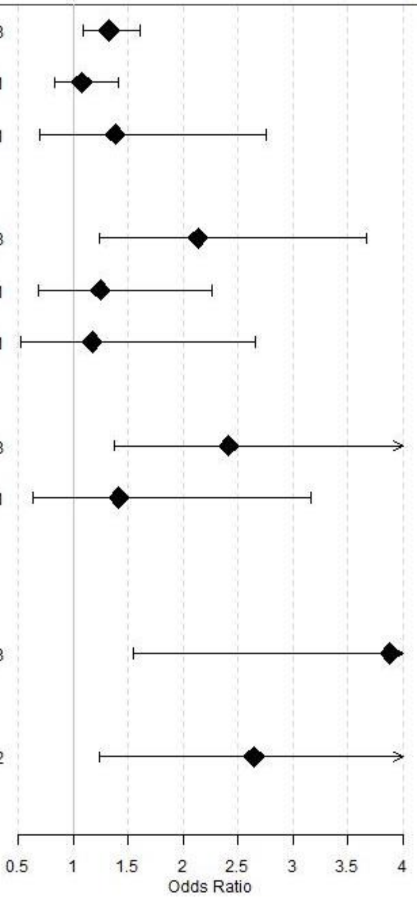

C) Female: Former smokers vs. never smokers

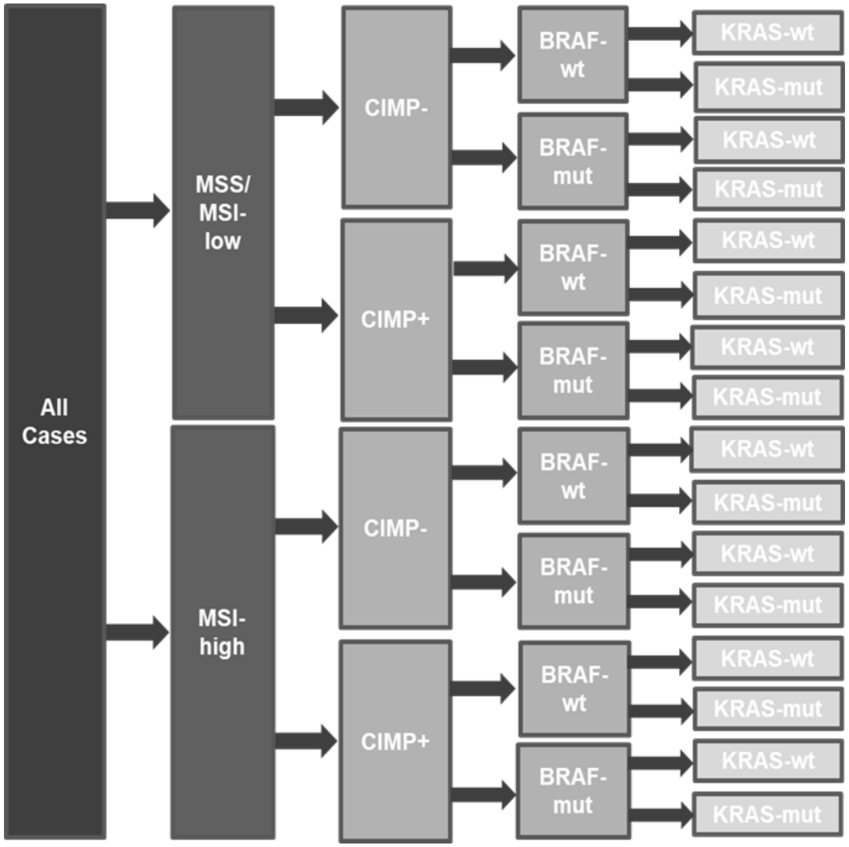

Subtypes	N Cases	OR	95% CI	p-value	p-difference
Type 4	410	1.17	(1.01,1.36)	3.53E-02	reference
Type 3	275	1.04	(0.87,1.23)	6.92E-01	1.71E-01
Type 6	37	1.29	(0.82,2.03)	2.72E-01	8.95E-01
Type 7	7				
Type 8	28	1.32	(0.81,2.13)	2.62E-01	9.65E-01
Type 9	29	1.19	(0.75,1.90)	4.60E-01	8.16E-01
Type 2	32	1.42	(0.88,2.29)	1.48E-01	7.79E-01
Type 10	0				
Type 5	23	0.76	(0.45,1.30)	3.17E-01	1.45E-01
Type 11	6	0.40	(0.16,1.00)	4.92E-02	1.05E-02
Type 12	10				
Type 13	1				
Type 14	28	1.47	(0.88,2.46)	1.42E-01	2.89E-01
Type 15	5				
Type 1	114	1.44	(1.10,1.90)	8.94E-03	2.51E-01
Type 16	1				

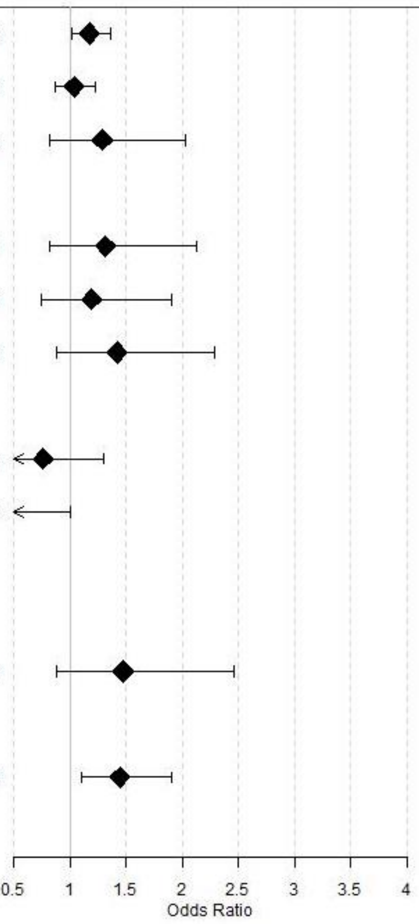

D) Current smokers vs. never smokers

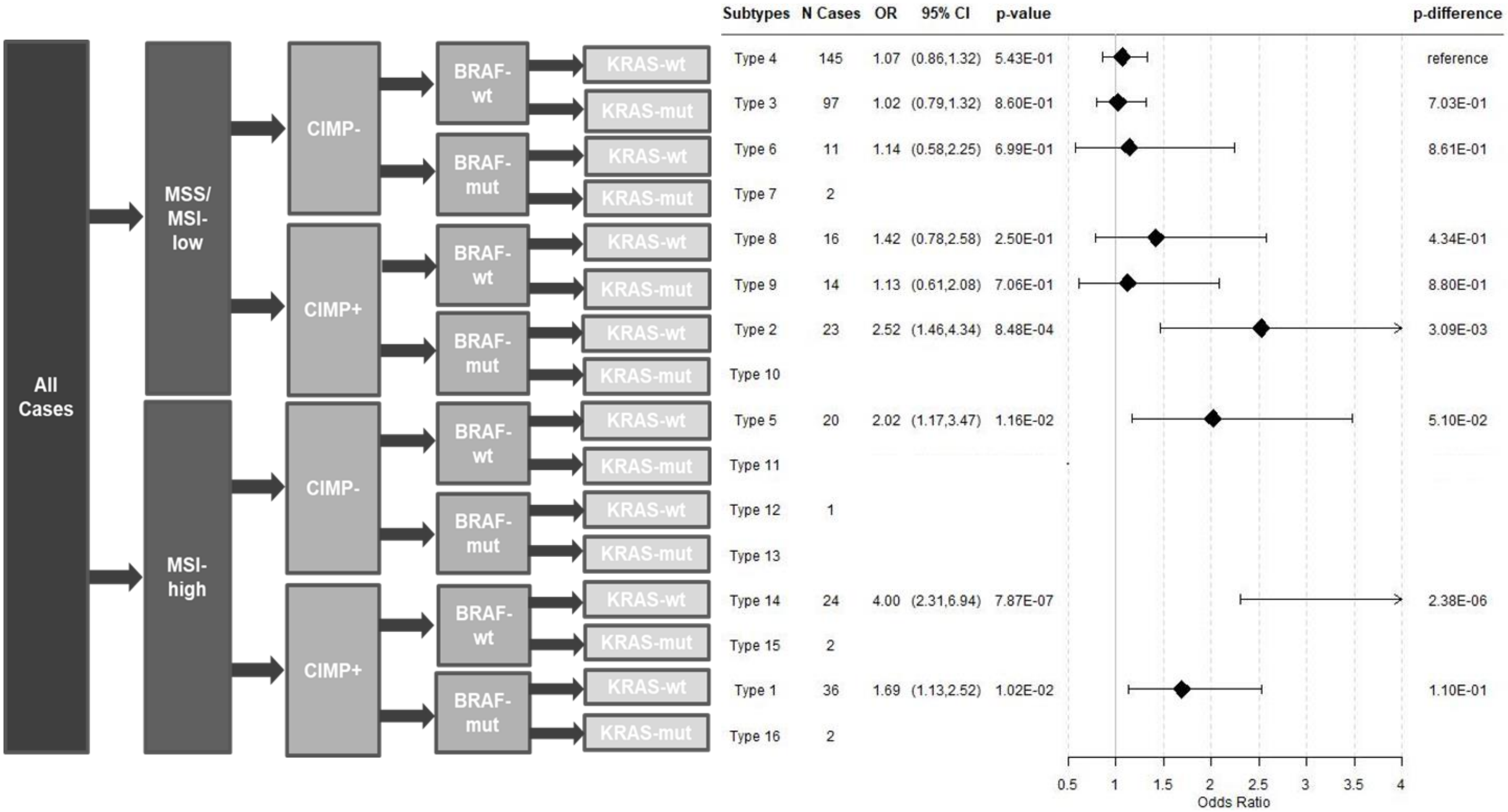

MSS: microsatellite stable; MSI: microsatellite instability; CIMP: CpG island methylation phenotype; wt: wild type; mut: mutated;OR: odds ratio; 95% CI: 95% confidence interval; Two-sided Wald test was used to calculate the P values from the case-control analysis (N controls = 5,987 for male and N controls = 5,675 for female) and case-only analysis (Pdifference). A Bonferroni corrected p-value threshold of $5.0x10^{-3}$ was used for both case-control and case-only analyses.

Supplementary Figure 5. Associations between smoking pack-years and risk of CRC subtypes defined by combined marker status, stratified by sex

A) Male

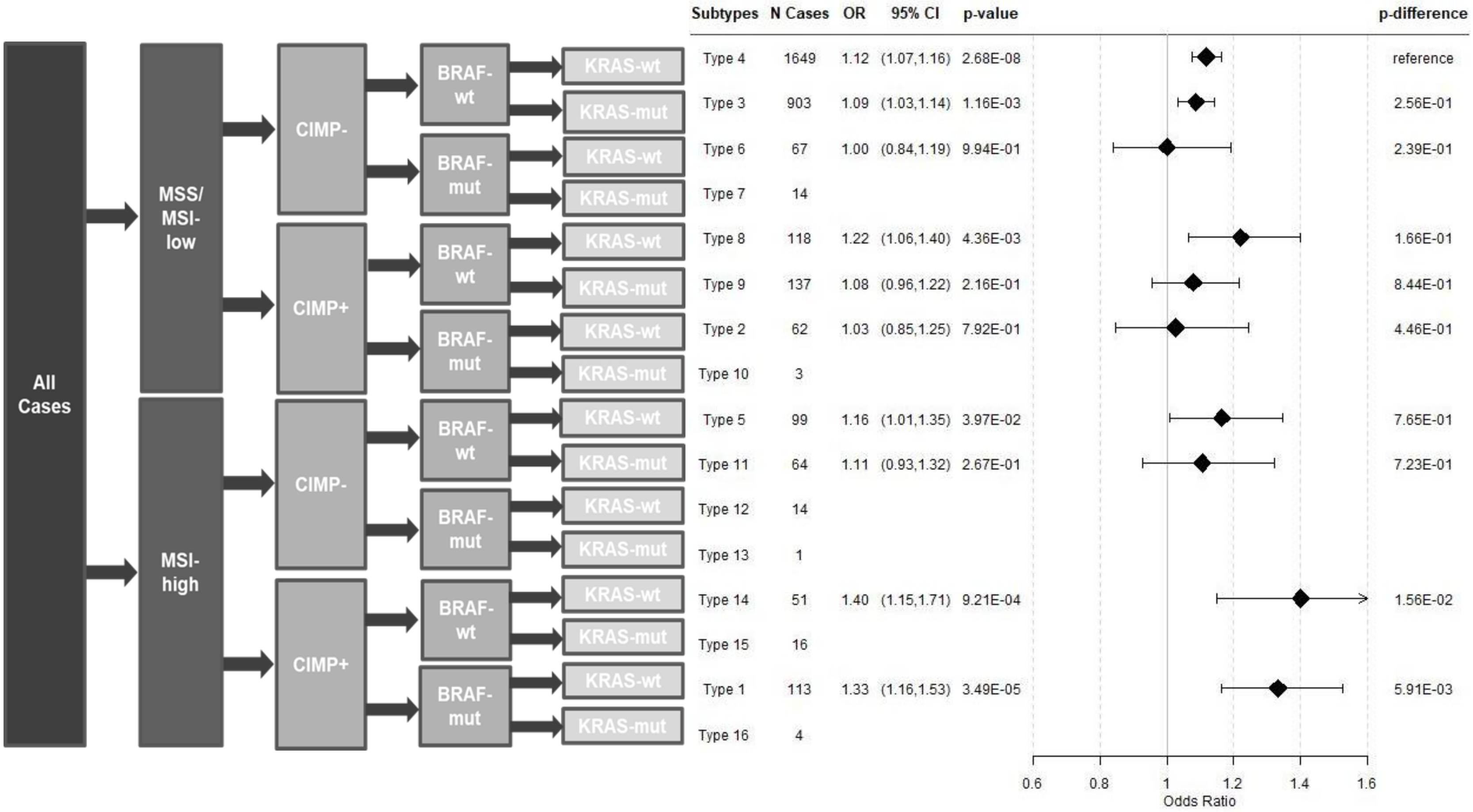

B) Female

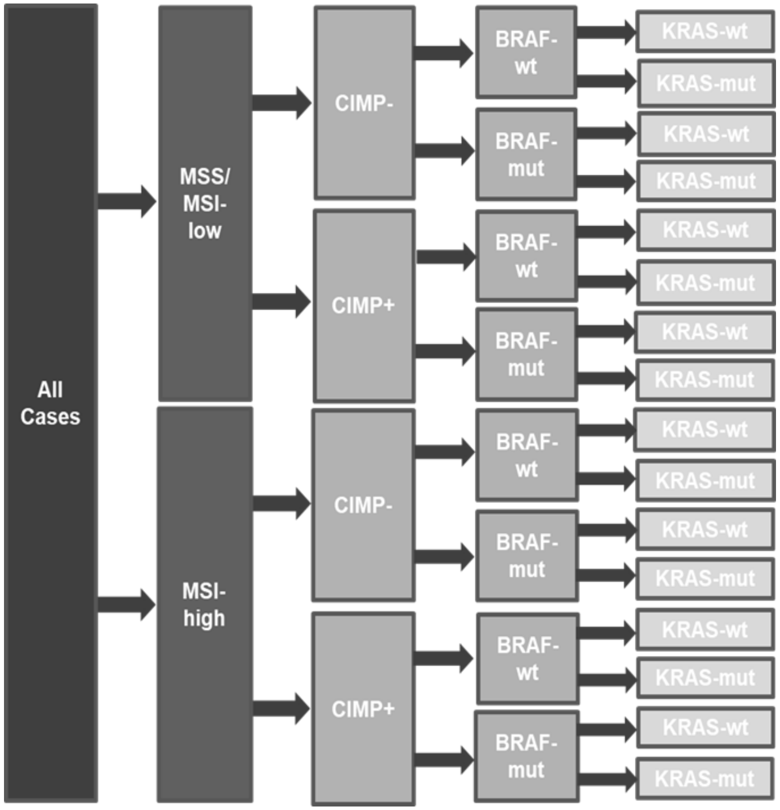

Subtypes	N Cases	OR	95% CI	p-value	p-difference
Type 4	410	1.17	(1.01,1.36)	3.53E-02	reference
Type 3	275	1.04	(0.87,1.23)	6.92E-01	1.71E-01
Type 6	37	1.29	(0.82,2.03)	2.72E-01	8.95E-01
Type 7	7				
Type 8	28	1.32	(0.81,2.13)	2.62E-01	9.65E-01
Type 9	29	1.19	(0.75,1.90)	4.60E-01	8.16E-01
Type 2	32	1.42	(0.88,2.29)	1.48E-01	7.79E-01
Type 10	0				
Type 5	23	0.76	(0.45,1.30)	3.17E-01	1.45E-01
Type 11	6	0.40	(0.16,1.00)	4.92E-02	1.05E-02
Type 12	10				
Type 13	1				
Type 14	28	1.47	(0.88,2.46)	1.42E-01	2.89E-01
Type 15	5				
Type 1	114	1.44	(1.10,1.90)	8.94E-03	2.51E-01
Type 16	1				

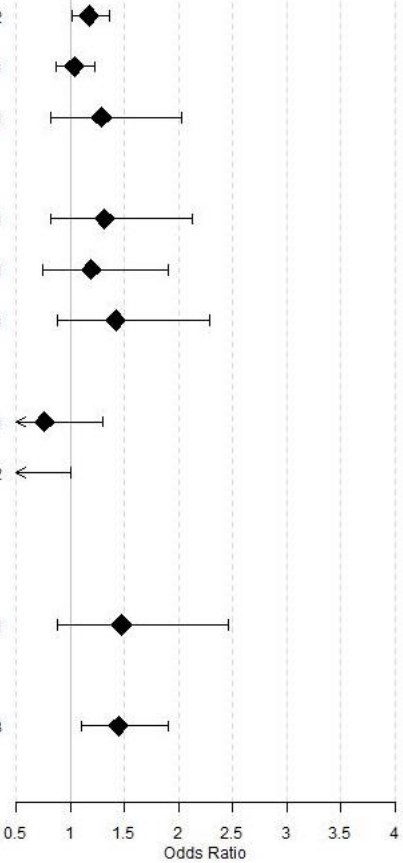

MSS: microsatellite stable; MSI: microsatellite instability; CIMP: CpG island methylation phenotype; wt: wild type; mut: mutated;OR: odds ratio; 95% CI: 95% confidence interval; Two-sided Wald test was used to calculate the P values from the case-control analysis (N controls = 5,987 for male and N controls = 5,675 for female) and case-only analysis (Pdifference). A Bonferroni corrected p-value threshold of $5.0x10^{-3}$ was used for both case-control and case-only analyses.

Supplementary Figure 6. Association between former and current smokers and risk of CRC subtypes defined by combined marker status, stratified by study design

A) Cohort: Former smokers vs. never smokers

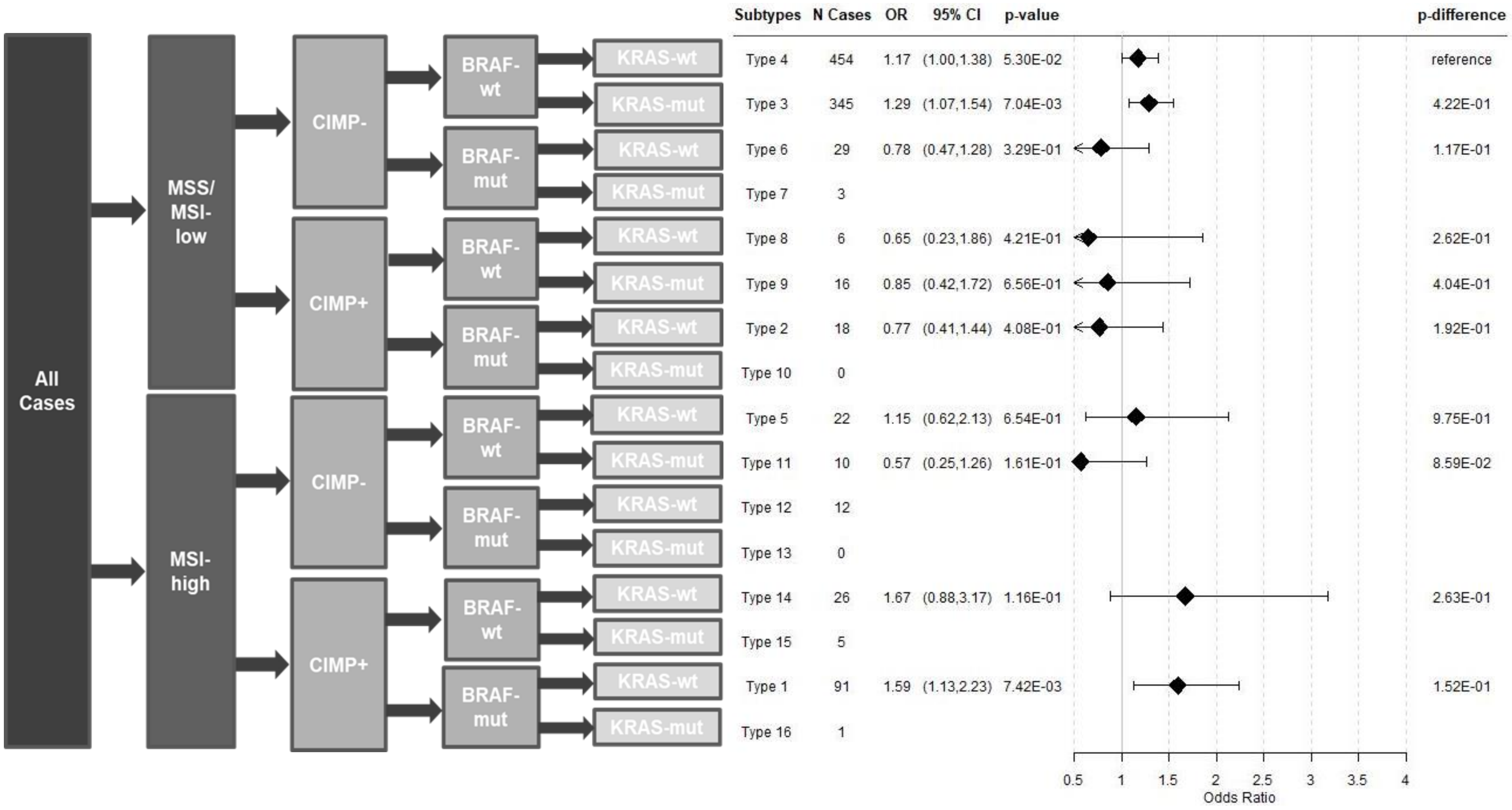

B) Cohort: Current smokers vs. never smokers

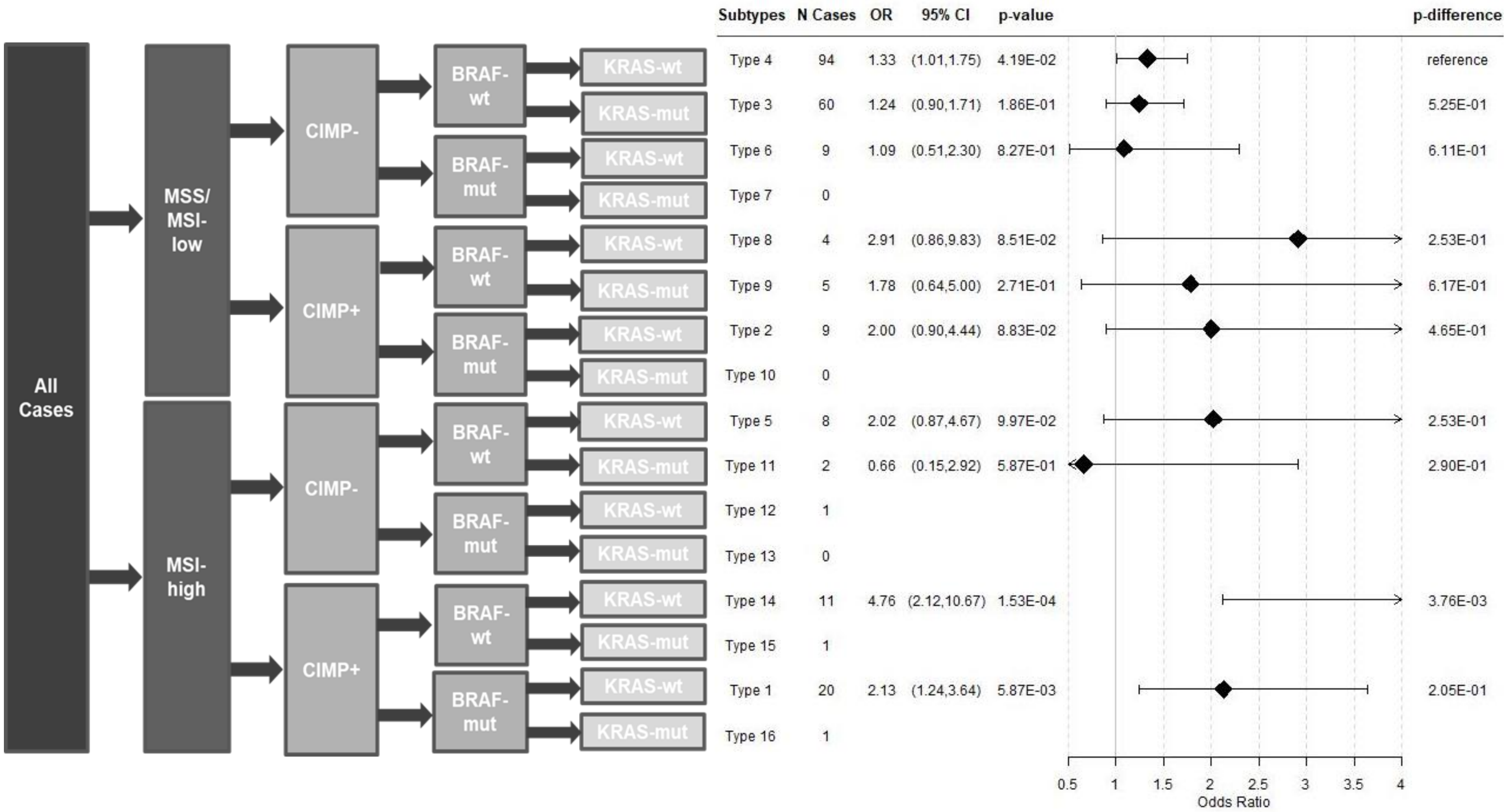

Subtypes	N Cases	OR	95% CI	p-value	p-difference
Type 4	94	1.33	(1.01,1.75)	4.19E-02	reference
Type 3	60	1.24	(0.90,1.71)	1.86E-01	5.25E-01
Type 6	9	1.09	(0.51,2.30)	8.27E-01	6.11E-01
Type 7	0				
Type 8	4	2.91	(0.86,9.83)	8.51E-02	2.53E-01
Type 9	5	1.78	(0.64,5.00)	2.71E-01	6.17E-01
Type 2	9	2.00	(0.90,4.44)	8.83E-02	4.65E-01
Type 10	0				
Type 5	8	2.02	(0.87,4.67)	9.97E-02	2.53E-01
Type 11	2	0.66	(0.15,2.92)	5.87E-01	2.90E-01
Type 12	1				
Type 13	0				
Type 14	11	4.76	(2.12,10.67)	1.53E-04	3.76E-03
Type 15	1				
Type 1	20	2.13	(1.24,3.64)	5.87E-03	2.05E-01
Type 16	1				

.

C) Case-control: Former smokers vs. never smokers

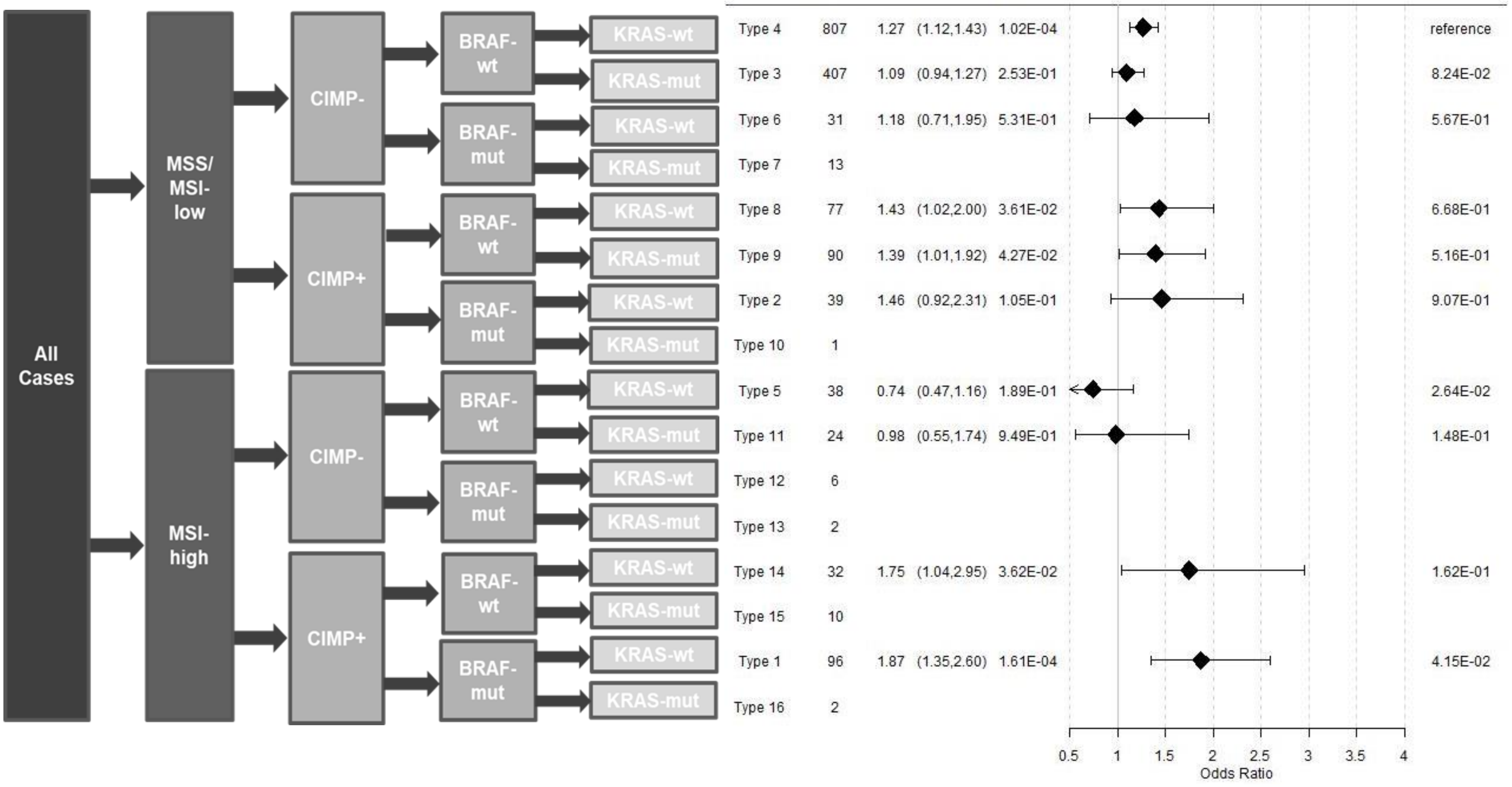

Subtypes	N Cases	OR	95% CI	p-value	p-difference
Type 4	807	1.27	(1.12,1.43)	1.02E-04	reference
Type 3	407	1.09	(0.94,1.27)	2.53E-01	8.24E-02
Type 6	31	1.18	(0.71,1.95)	5.31E-01	5.67E-01
Type 7	13				
Type 8	77	1.43	(1.02,2.00)	3.61E-02	6.68E-01
Type 9	90	1.39	(1.01,1.92)	4.27E-02	5.16E-01
Type 2	39	1.46	(0.92,2.31)	1.05E-01	9.07E-01
Type 10	1				
Type 5	38	0.74	(0.47,1.16)	1.89E-01	2.64E-02
Type 11	24	0.98	(0.55,1.74)	9.49E-01	1.48E-01
Type 12	6				
Type 13	2				
Type 14	32	1.75	(1.04,2.95)	3.62E-02	1.62E-01
Type 15	10				
Type 1	96	1.87	(1.35,2.60)	1.61E-04	4.15E-02
Type 16	2				

D) Case-control: Current smokers vs. never smokers

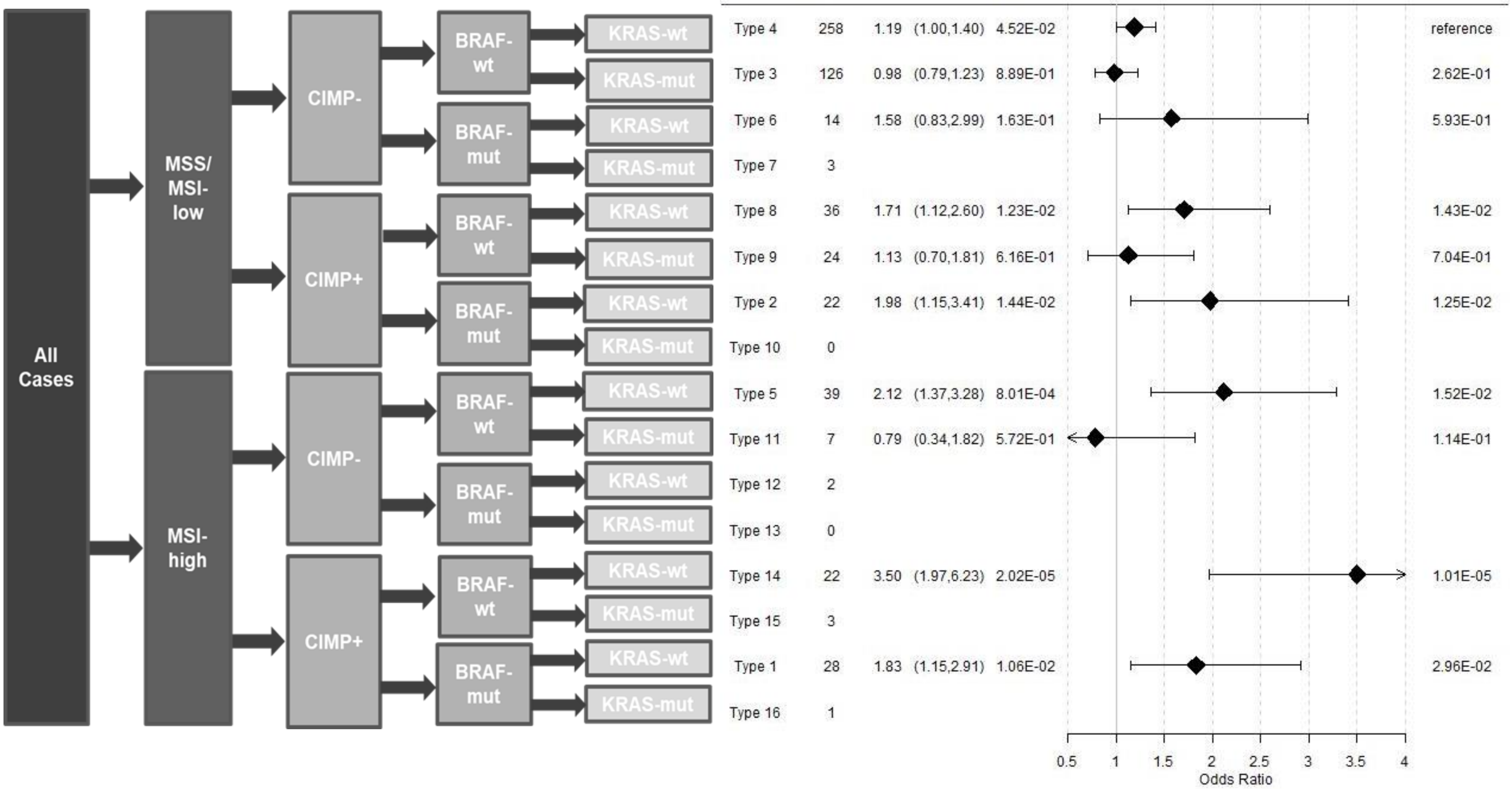

Subtypes	N Cases	OR	95% CI	p-value	p-difference
Type 4	258	1.19	(1.00,1.40)	4.52E-02	reference
Type 3	126	0.98	(0.79,1.23)	8.89E-01	2.62E-01
Type 6	14	1.58	(0.83,2.99)	1.63E-01	5.93E-01
Type 7	3				
Type 8	36	1.71	(1.12,2.60)	1.23E-02	1.43E-02
Type 9	24	1.13	(0.70,1.81)	6.16E-01	7.04E-01
Type 2	22	1.98	(1.15,3.41)	1.44E-02	1.25E-02
Type 10	0				
Type 5	39	2.12	(1.37,3.28)	8.01E-04	1.52E-02
Type 11	7	0.79	(0.34,1.82)	5.72E-01	1.14E-01
Type 12	2				
Type 13	0				
Type 14	22	3.50	(1.97,6.23)	2.02E-05	1.01E-05
Type 15	3				
Type 1	28	1.83	(1.15,2.91)	1.06E-02	2.96E-02
Type 16	1				

MSS: microsatellite stable; MSI: microsatellite instability; CIMP: CpG island methylation phenotype; wt: wild type; mut: mutated;OR: odds ratio; 95% CI: 95% confidence interval; Two-sided Wald test was used to calculate the P values from the case-control analysis (N controls = 3,184 for cohort and N controls = 8,478 for case-control) and case-only analysis (Pdifference). A Bonferroni corrected p-value threshold of 5.0×10^{-3} was used for both case-control and case-only analyses.

Supplementary Figure 7. Associations between smoking pack-years and risk of CRC subtypes defined by combined marker status, stratified by study design

A) Cohort

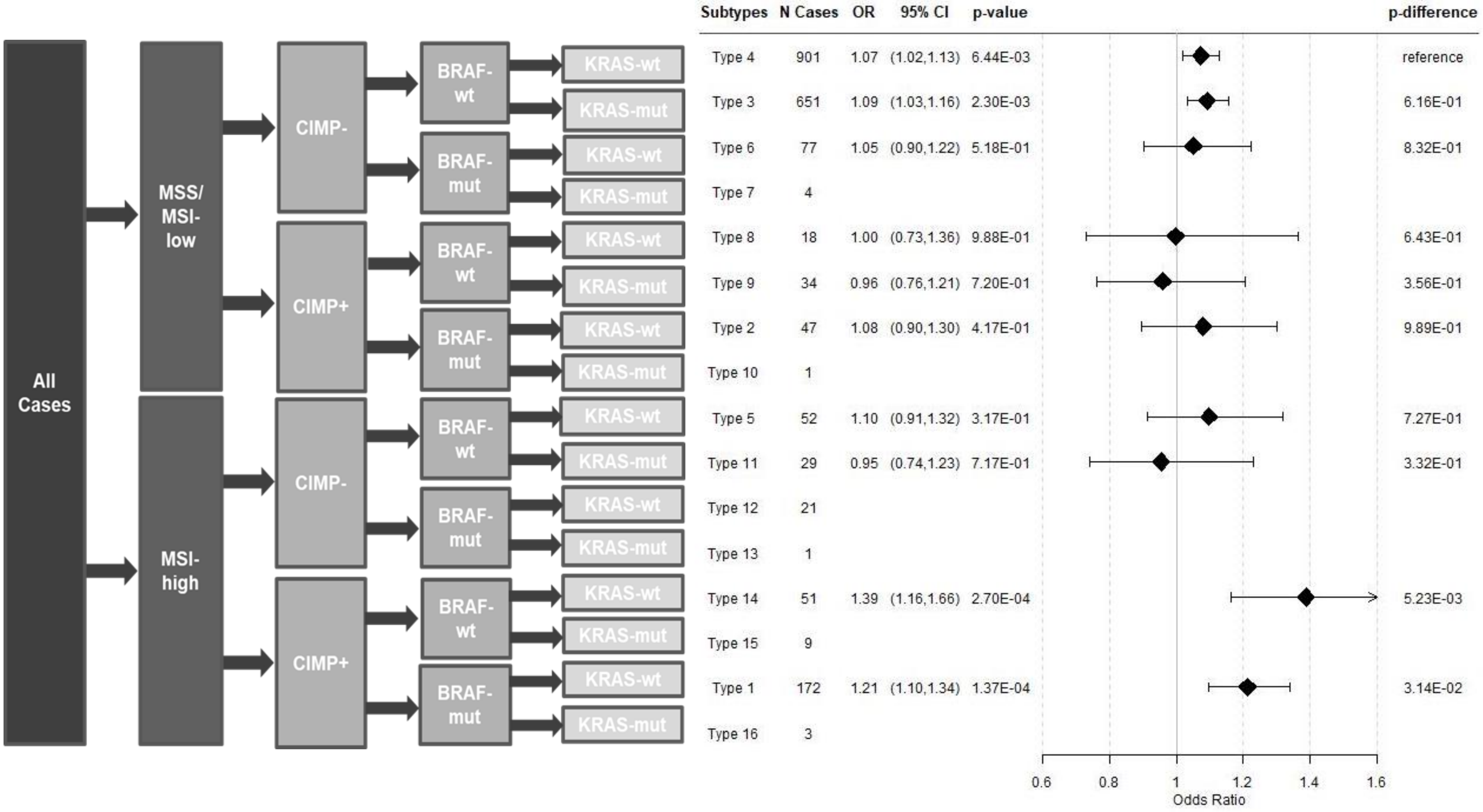

B) Case-control

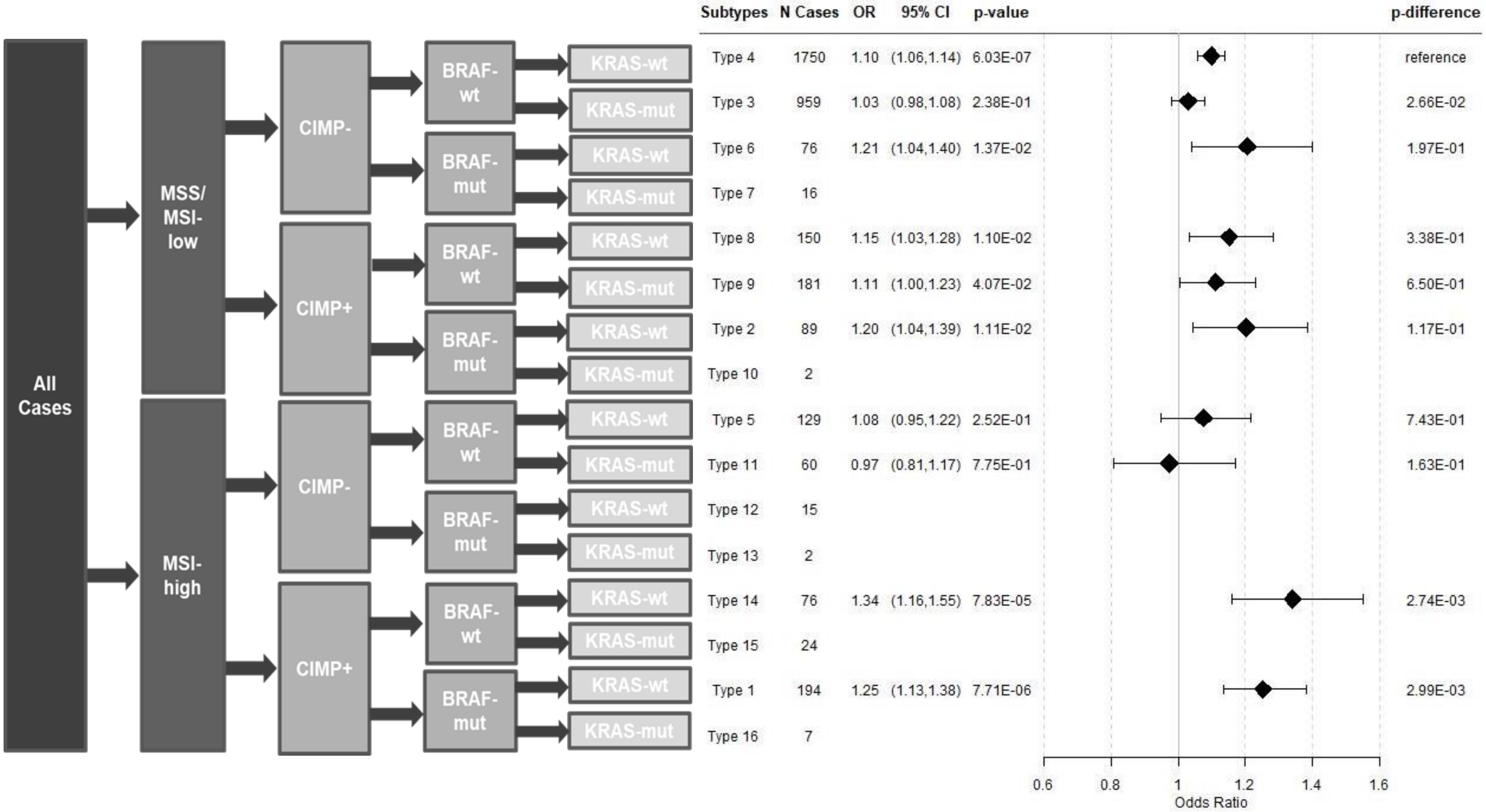

Subtypes	N Cases	OR	95% CI	p-value	p-difference
Type 4	1750	1.10	(1.06,1.14)	6.03E-07	reference
Type 3	959	1.03	(0.98,1.08)	2.38E-01	2.66E-02
Type 6	76	1.21	(1.04,1.40)	1.37E-02	1.97E-01
Type 7	16				
Type 8	150	1.15	(1.03,1.28)	1.10E-02	3.38E-01
Type 9	181	1.11	(1.00,1.23)	4.07E-02	6.50E-01
Type 2	89	1.20	(1.04,1.39)	1.11E-02	1.17E-01
Type 10	2				
Type 5	129	1.08	(0.95,1.22)	2.52E-01	7.43E-01
Type 11	60	0.97	(0.81,1.17)	7.75E-01	1.63E-01
Type 12	15				
Type 13	2				
Type 14	76	1.34	(1.16,1.55)	7.83E-05	2.74E-03
Type 15	24				
Type 1	194	1.25	(1.13,1.38)	7.71E-06	2.99E-03
Type 16	7				

MSS: microsatellite stable; MSI: microsatellite instability; CIMP: CpG island methylation phenotype; wt: wild type; mut: mutated;OR: odds ratio; 95% CI: 95% confidence interval; Two-sided Wald test was used to calculate the P values from the case-control analysis (N controls = 3,184 for cohort and N controls = 8,478 for case-control) and case-only analysis (Pdifference). A Bonferroni corrected p-value threshold of 5.0×10^{-3} was used for both case-control and case-only analyses.
